# Fatigue Damage Mechanisms and Complementary Non-Destructive Testing of Thin-Walled Structures with Various Composite Material Lay-Ups

**DOI:** 10.3390/ma19214726

**Published:** 2026-11-06

**Authors:** Patryk Różyło, Jakub Paśnik, Adam Stawiarski, Małgorzata Chwał, Paweł J. Romanowicz, Marek Barski

**Affiliations:** 1 Department of Machine Design and Mechatronics, Faculty of Mechanical Engineering, Lublin University of Technology, Nadbystrzycka 36, 20-618 Lublin, Poland; j.pasnik@pollub.pl; 2 Department of Machine Design and Composite Structures, Faculty of Mechanical Engineering, Cracow University of Technology, al. Jana Pawła II 37, 31-864 Kraków, Poland; adam.stawiarski@pk.edu.pl (A.S.); malgorzata.chwal@pk.edu.pl (M.C.); pawel.romanowicz@pk.edu.pl (P.J.R.); marek.barski@pk.edu.pl (M.B.)

**Keywords:** fatigue strength, NDT methods, damage initiation, various lay-ups of composite

## Abstract

**Highlights:**

- The fatigue response of thin-walled composite structures was investigated.
- The effect of composite layer configuration on fatigue behaviour was tested.
- The phenomenon of failure was analysed using non-destructive testing techniques.

**Abstract:**

This paper focuses on the fatigue response of composite structures, considering complementary (different) non-destructive testing (NDT) techniques. The research concerns thin-walled profiles with a notch and several different composite material layer arrangements (composite lay-ups). The research determined the influence of the layer arrangement on fatigue strength and laminate damage forms. A fatigue testing device, passive thermography to assess thermal changes, acoustic emission to detect damage initiation and digital microscopy to analyse damage forms were utilised in the research. The correct parameters for fatigue testing were determined during the test trials to ensure that the test specimens did not lose their load-carrying capacity. The research aimed to analyse the phenomenon of composite failure during fatigue testing in quantitative and qualitative aspects. The study only considered fatigue response in the context of permissible operating loads—excluding tests that took into account the total failure of the specimens.

## 1. Introduction

Fatigue response is one of the crucial aspects of materials mechanics, directly affecting the safety and durability of structures. Under cyclic loading, a material gradually accumulates damage, which leads to a decrease in its load-carrying capacity, even for stresses that are lower than the yield strength. Since the beginning of the 20th century, classical fatigue testing of metals [1,2,3,4,5] has contributed to the development and experimental validation of mathematically mature models for describing fatigue crack propagation in homogeneous materials. In recent years, there has been a rapid development of research on composites, particularly those with a polymer matrix [6,7,8,9]. With the advancement of materials engineering, the rapid growth of fibrous composites has emerged, which today constitute one of the basic groups of materials in aviation, automotive, wind energy, and defence industries [6]. With their optimal strength-to-weight ratio and the possibility to tailor their mechanical properties to specific applications, these materials can significantly reduce structural weight while maintaining high fatigue strength. Nevertheless, fatigue in fibre-reinforced polymer (FRP) composites remains a highly complex and under-explored problem, requiring an interdisciplinary approach involving mechanics, materials science, as well as NDT methods [8].

Composite materials are characterised by the anisotropy of their mechanical properties and complex damage morphology. As opposed to metallic materials, where crack initiation and propagation are the dominant mechanisms, a wide range of local processes occur in composites. Depending on the geometry of the test profiles, the type of reinforcement, the orientation of the fibre, the environmental conditions, and the mode of loading, different types of microdamage, such as matrix microcracks, interlaminar delamination, fibre–matrix debonding or fibre breakage, may occur simultaneously in the composite [10,11,12,13,14]. As fatigue progresses, these damages interact with each other—the propagation of microcracks in the matrix leads to local delamination of the laminate, which, in turn, leads to localised stress concentrations and further damage to the fibrous layups.

The fatigue models developed for FRP laminates are based on both an empirical approach [13,15] as well as energy and micromechanical methods [16,17]. A number of studies indicate that the degradation of the elastic modulus and the increase in deformation are good indicators of damage progression [18,19]. Experimental studies [20,21] have shown that local stress concentration and irregular fibre orientation have the strongest contribution to damage development. High R-ratio values promote delamination [22] and cyclic loads with variable direction lead to defect accumulation in oblique planes [23]. Predictive models [24,25] combine elements of cohesion zone theory and strain energy analysis, enabling the description of the failure process of layered composites under long-term loading.

The fatigue damage in composites usually initiates in areas of local stress concentration: near air pockets, manufacturing defects, bonding failures, or fibres with incorrect orientation. Cyclic stresses cause microcracks to form in the matrix, which over time lead to fibres separating from the matrix (debonding) and to delamination propagation between layers. Studies [8,16] have shown that the combination of mechanical and thermal effects plays a key role in the degradation process—local heating of the specimen (the so-called self-heating effect) significantly accelerates the development of microdamage. In addition, environmental exposure, particularly humidity and temperature, leads to interfacial degradation by altering fibre-matrix adhesion [26]. Consequently, the analysis of fatigue mechanisms in composites requires both an experimental and a numerical approach that takes into account the influence of the microscopic morphology of the material.

In modern fatigue studies of composites, NDT methods are increasingly combined with conventional cyclic loading experiments. This integration enables real-time monitoring of the phenomenon of damage, thereby improving fatigue life prediction and model validation. The most commonly used techniques are infrared thermography (IRT), digital image correlation (DIC), acoustic emission (AE) and computed tomography (CT/μCT).

IRT involves measuring surface temperature variations caused by internal energy dissipation during cyclic loading. It enables the detection of localised heat sources associated with delamination due to friction, matrix cracking or fibre–matrix debonding. In paper [27], the authors demonstrated that IRT can effectively determine critical stress levels and predict fatigue thresholds in polymeric composites by analysing self-heating patterns. DIC is a real-time, non-contact optical method that captures surface displacements and strains. For composite laminates, DIC enables the visualisation of strain localisation, early crack initiation and interlaminar shear deformation. Authors of [28] in their studies have integrated DIC data with finite element (FE) models to predict delamination initiation under cyclic loads. AE monitors detect elastic waves generated by microcracking, fibre fracture or delamination [29,30]. It enables the classification of damage mechanisms and the identification of the current stage of damage during fatigue cycling [23]. The author of [31] analysed AE signal characteristics in laminated composites and demonstrated that energy distribution parameters strongly correlate with fatigue degradation stages. X-ray tomography, or computer tomography, provides three-dimensional visualisation of internal structures, enabling the detection of voids, cracks and delaminations within the composite volume. CT was used by the authors of [32] to evaluate the structural quality of curved CFRP laminates, demonstrating its ability to assess damage in composite laminates. Feasibility and application value of using non-destructive techniques to dynamically monitor interlayer fatigue damage is confirmed in experimental studies of different materials [33,34,35]. The use of multiple diagnostic techniques allows for a more complete understanding of complex material degradation processes [36,37,38,39].

The results reported by [16] confirm that the fatigue resistance of laminates is strongly dependent on the configuration of the layers and the orientation of the fibres, as well as on the type of cyclic loading. This paper remains an important reference in research on the fatigue and damage mechanics of fibre-reinforced composites, particularly in the development of strength criteria and the prediction of the fatigue life of composite structures. The work [20] proves the effectiveness of the residual strength model in assessing the durability of notched reinforced laminates, while research [21] combines experimentation with numerical analysis using advanced damage models (PFA). Panasiuk and Dudzik [31] used acoustic emission to identify stages of destruction, and the authors of [27,37] proved the usefulness of thermography in fatigue diagnostics.

This type of interdisciplinary approach combines numerical methods, experimental measurements, and non-destructive diagnostics, which forms the basis for innovative research on the durability of fibre composites in Poland and worldwide [17,36,40].

Fatigue testing of fibrous composites is one of the most dynamically developing areas of materials mechanics. The multi-stage nature of damage and the coexistence of various degradation mechanisms mean that classic approaches based on S–N curves are insufficient. The integrated use of modern diagnostic methods, such as thermography, DIC, AE, and computed tomography, allows for a complete overview of the fatigue process, and the solutions developed by researchers, combining experimental measurements with numerical simulations, represent an innovative direction in the development of modern composite research [41,42,43,44,45,46].

Although previous studies have demonstrated the potential of combining non-destructive testing techniques to monitor damage in composite materials, the value of such an approach depends on the specific research question and on how the complementary measurements are interpreted. In the present study, the focus is on comparing the damage-related responses of CFRP laminates with different stacking sequences under cyclic tensile loading using acoustic emission (AE) and thermal monitoring. The above-mentioned techniques provide complementary information: AE captures transient activity associated with damage-related events, while thermal monitoring characterises changes in the specimen’s thermal response during loading.

## 2. Materials and Methods

### 2.1. Subject of Study

**Figure 1:**
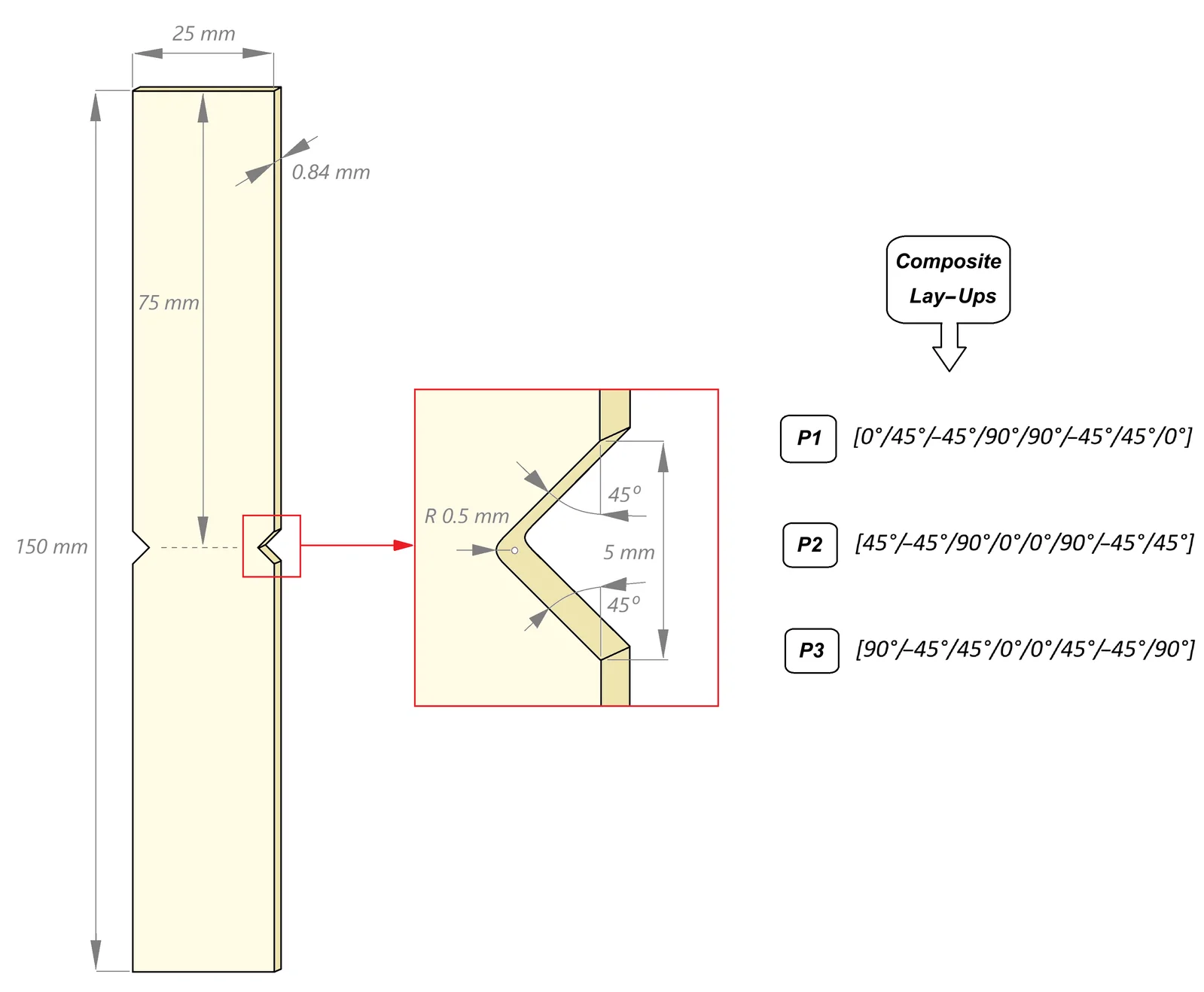
Geometric parameters and laminate lay-ups of the test specimens.

The research investigated the fatigue response of thin-walled composite structures manufactured from CFRP laminates. The test specimens were manufactured using the autoclave technique by an external company—WIT-Composites (Krasienin, Poland). As shown in Figure 1, the test specimens had uniform geometric parameters. The test specimens featured different composite laminate lay-ups, resulting from variations in the arrangement and orientation of individual plies. The study considered three laminate layer arrangements, as shown in Figure 1.

**Figure 2:**
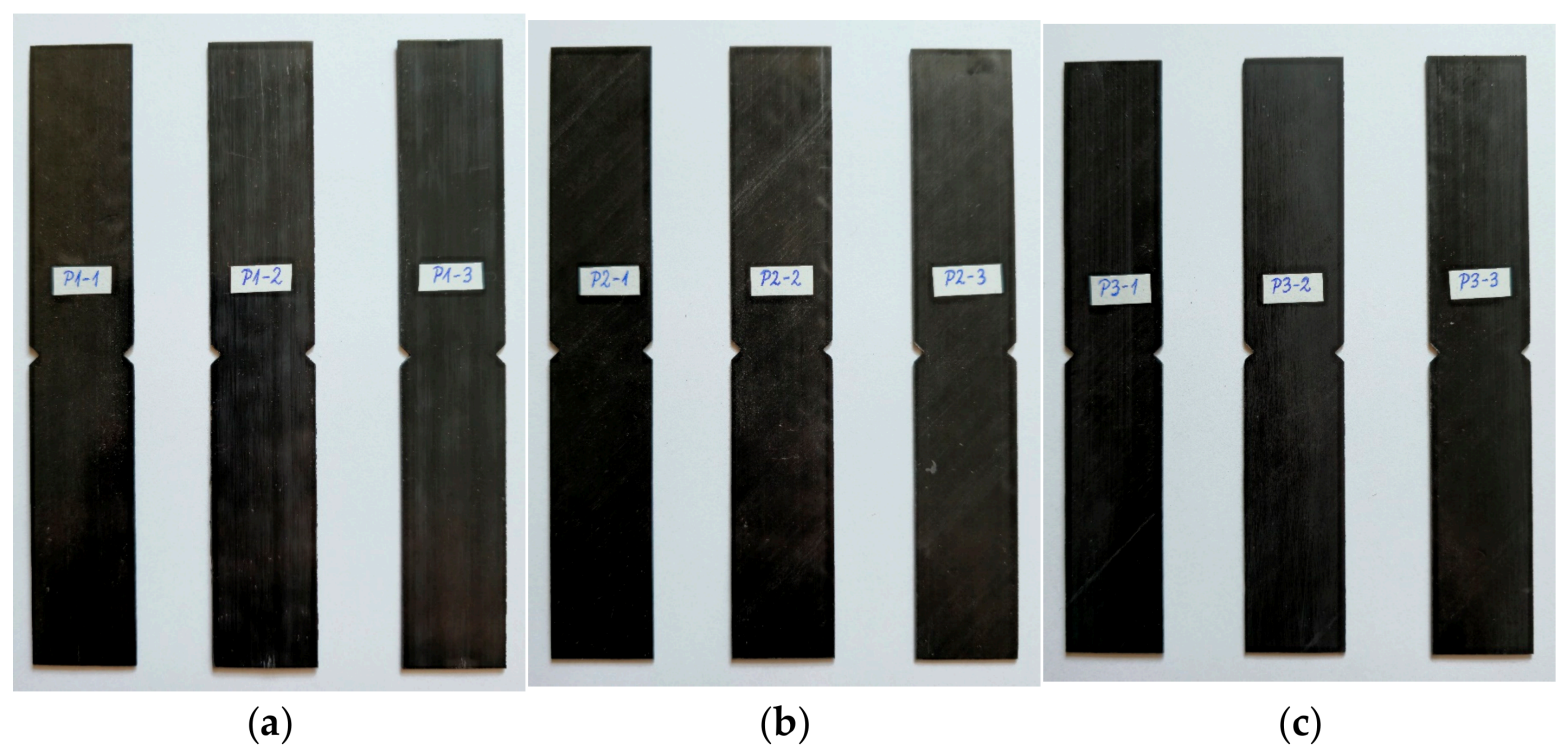
Test specimens for experimental testing: (**a**) P1-type specimens; (**b**) P2-type specimens; (**c**) P3-type specimens.

A total of nine test specimens were produced for testing: three specimens for each composite lay-up: P1 [0°/45°/−45°/90°]_s_, P2 [45°/−45°/90°/0°]_s_, P3 [90°/−45°/45°/0°]_s_. All composite material layups were made up of the same number of 0°, 45° and 90° layers, arranged in a different sequence. A common feature was the symmetrical arrangement of the composite layers. The test specimens also featured an identical notch, as shown in Figure 1. In the case of the P1 composite lay-up, the specimens were labelled P1-1, P1-2 and P1-3. The specimens for the other two composite lay-ups were labelled similarly. Figure 2 shows the specimens used in the experimental tests.

**Figure 3:**
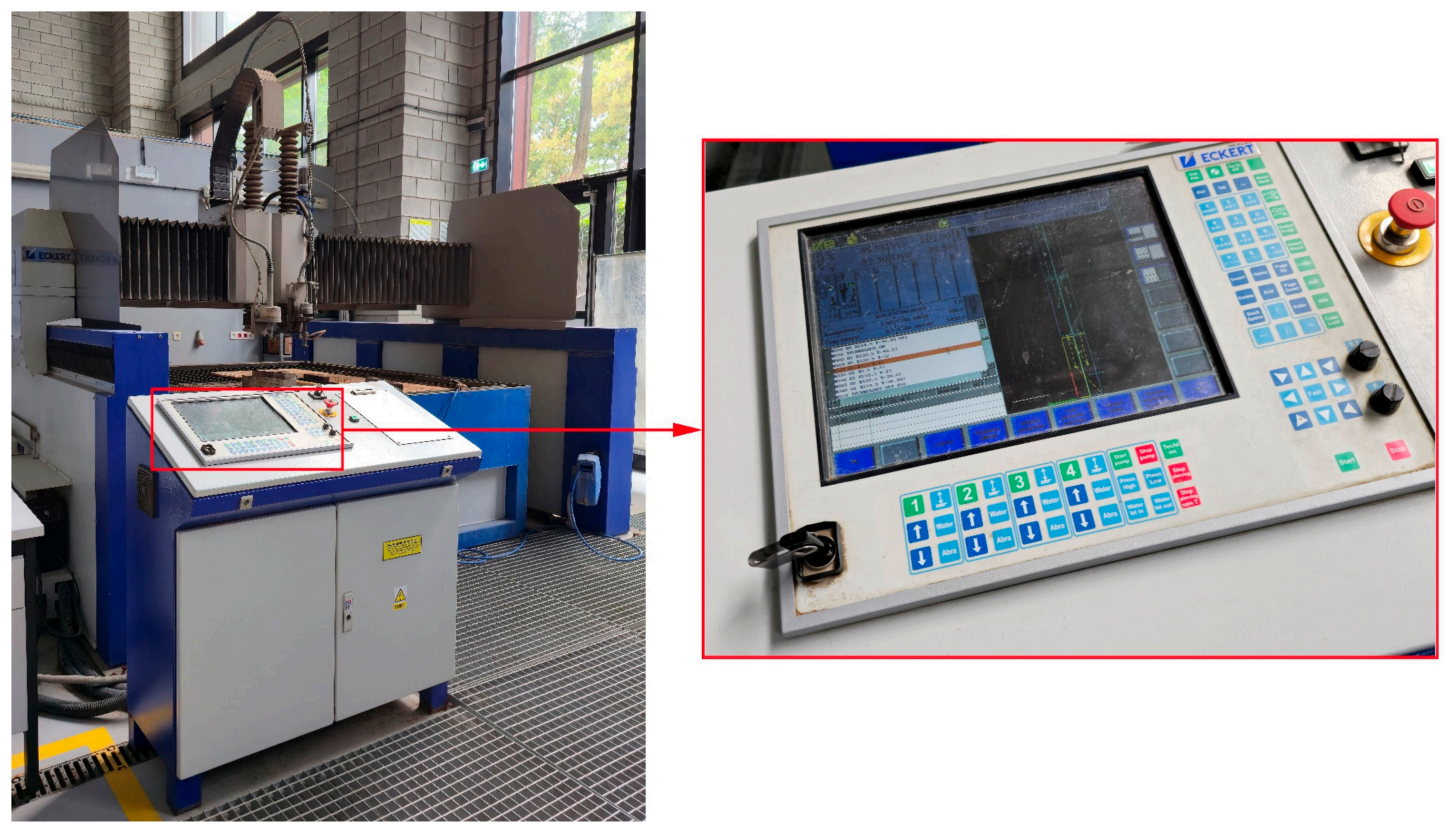
A machine for cutting through materials using a stream of water at high pressure.

The specimens were carefully prepared in accordance with the geometric parameters. For this purpose, water jet cutting technology was used. The samples were cut from larger composite components that were not used for other research purposes [47,48,49]. A jet with a diameter of 1 mm was used for the water jet cutting process. The waterjet cutting machine is shown in Figure 3.

The material properties were determined in accordance with the applicable material-specific standards. The properties of carbon fibre reinforced polymer (CFRP) composite material were specified using several standards: PN-EN ISO 527–5 (of 2010)—tensile test, PN-EN ISO 14126 (of 2002)—compression test, as well as PN-EN ISO 14129 (of 2000)—shear test. The parameters of the composite, which were determined in previous research activities, have been presented in other publications [48,49]. A detailed methodology for the experimental characterisation of the mechanical properties of CFRP composites using electrical resistance strain gauges was previously reported in the literature [50]. Publication [50] outlines a step-by-step approach to determining the required material parameters for carbon-epoxy composite (CFRP). Table 1 shows the material parameters determined on the basis of the above-mentioned standards.

The parameters presented in Table 1 represent average values of experimentally determined material properties. The accuracy and correctness of determining the characteristics of composites are confirmed by previously published papers, in which stability and failure tests were conducted on compressed, thin-walled composite structures (with high agreement between experimental tests and FEM simulations) [48,49]. Further details on the production of composite structures can be found in a prior scientific publication [51].

### 2.2. Experimental Research Methodology

#### 2.2.1. Fatigue Test Setup and Loading Conditions

Interdisciplinary research techniques were employed in the experimental research. The main apparatus was a strength testing machine that enabled fatigue testing. Fatigue tests were conducted as low-cycle tests. Fatigue tests were performed using an MTS Landmark 370 servo-hydraulic testing machine (MTS Systems Corporation, Eden Prairie, MN, USA) [52]. The fatigue testing system had a maximum load capacity of 100 kN. The tests were performed using dedicated testing equipment, with the specimens secured in hydraulic grips (model 647.10A). The clamping force applied to the test specimens was 2000 Psi (13.79 MPa). A force load was applied during testing and monitored using a dedicated MTS force transducer controller—model 661.20H-03 (MTS Systems Corporation, Eden Prairie, MN, USA). The tests were recorded using MTS Flex Test™ 40 Station Manager—specialised software that enables the recording of test trials and the subsequent analysis of results. The main aims of the study were to investigate fatigue damage mechanisms in thin-walled structures in the low-cycle fatigue range using different non-destructive testing (NDT) techniques. Because of this, the maximal load was set to equal 80% of the critical static load. To avoid the risk of specimen compression during the test, a fatigue test with the stress ratio *R* = 0.1 was employed, which is typically used in fatigue studies. The frequency was selected so that temperature changes from structural degradation could be observed with a thermal imaging camera, while ensuring the excitation frequency did not heat the samples. The test was stopped after 10,000 cycles. This is a typical limit for low-cycle fatigue. It allowed for the observation of various forms of structural degradation without the risk of complete failure of the specimens.

**Figure 4:**
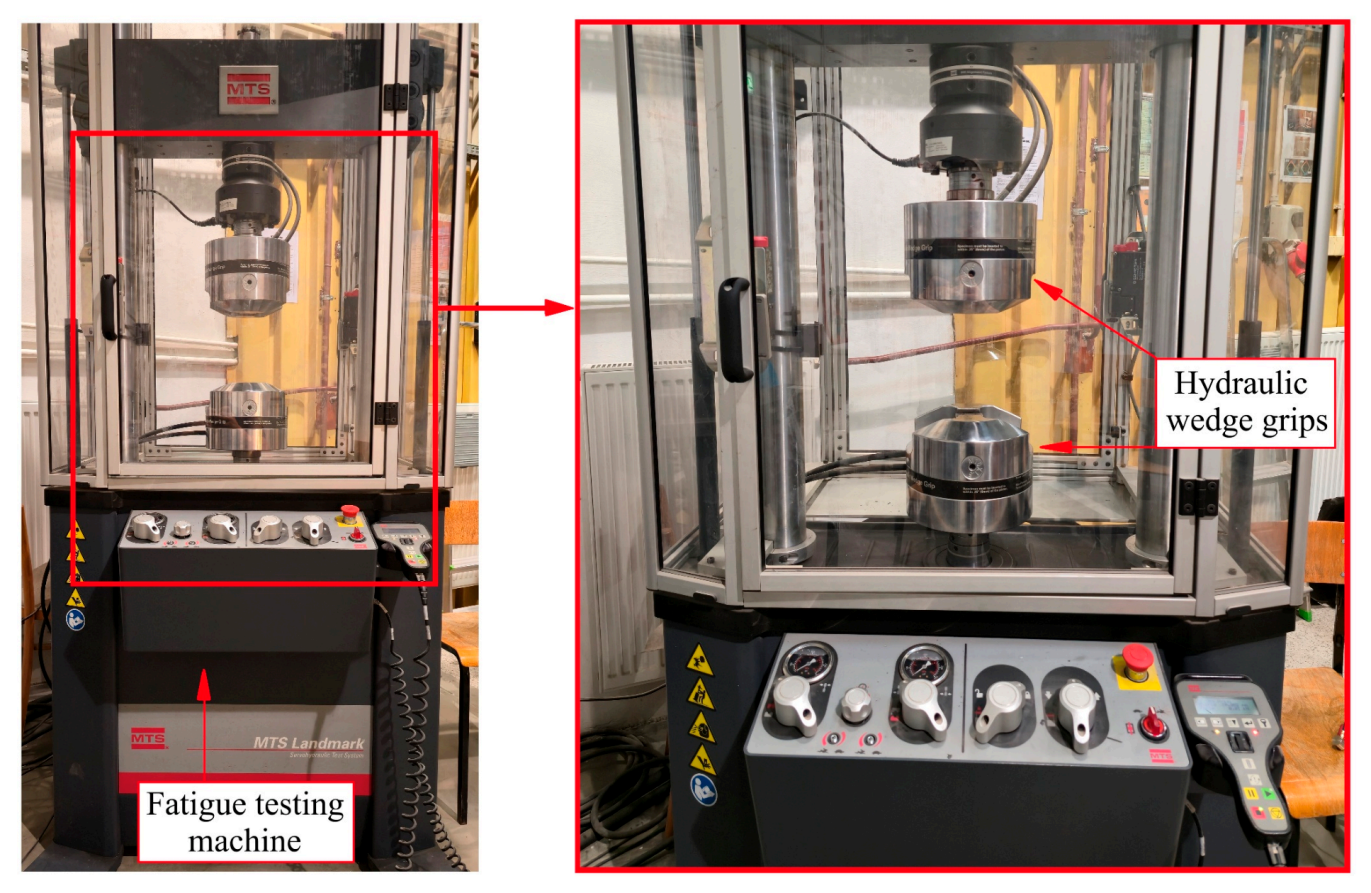
A machine for conducting fatigue tests.

This enabled the selection of the correct parameters for the main fatigue tests. To correctly perform a series of fatigue tests for research purposes, the following constant parameters were estimated: *F*_max_ = 10,000 N (maximum load); *F*_min_ = 1000 N (minimum load); *F*_m_ = 5500 N (average load); *F*_a_ = 4500 N (amplitude load); *f* = 10 Hz (frequency); *R* = 0.1 (amplitude coefficient); *N* = 10,000 (number of cycles). Prior to fatigue testing, the specimens were subjected to an initial tensile preload corresponding to the mean load, *F*_m_, at a loading rate of 10 kN/min. Fatigue loading was applied in the form of a sinusoidal force waveform, with each fatigue cycle consisting of a tensile loading phase followed by a relaxation phase. All tests were performed under the same loading and environmental conditions in a laboratory with steady-state ambient conditions. The fatigue tests were conducted with particular attention to the initiation of damage within the composite material, defined as the appearance of the first detectable signs of damage, while maintaining the load-carrying capacity of the structure. Figure 4 demonstrates a fatigue testing machine.

During fatigue testing, the specimens were clamped in the hydraulic grips using an identical and consistent clamping configuration. A distance of 45 mm was measured from the top and bottom edges of each test specimen. The specimens were fixed permanently within these measured areas. The research activities included studying the impact of composite lay-up on fatigue strength, as well as considering the possibility of using combined non-destructive testing methods to assess damage caused during fatigue tests.

#### 2.2.2. Definition of Equivalent Structural Stiffness Parameter

In fatigue testing, the main parameter recorded was specimen elongation, which increased in real time. This was compared with temperature increases (gradient) from thermography and energy signals from acoustic emission. An additional parameter analysed was the equivalent structural stiffness *K* of the specimen, which is defined as the ratio of the amplitude of the applied load (*F*_max_ − *F*_min_) and the specimen’s elongation (*u*_max_ − *u*_min_). The maximum elongation of the specimen, *u*_max_, was determined at the same point in time at which the maximum force *F*_max_ occurred. Analogously, the minimum elongation of the specimen, *u*_min_, was measured at the point at which the specimen was tensioned by the force *F*_min_. To reduce the influence of the sensor sampling frequency and limit the number of data points, while simultaneously providing the ability to capture extreme loads (minimum and maximum forces and elongations), the equivalent stiffness *K* was evaluated over a predefined range of fatigue cycles between *n*–*m* interval. It was assumed that the *n*–*m* interval was exactly 75 cycles. The equivalent stiffness of the specimen was calculated using the following relationship:

(1) $$K\left(n-m\right)=\frac{F_{\max}\left(n-m\right)-F_{\min}\left(n-m\right)}{u_{\max}\left(n-m\right)-u_{\min}\left(n-m\right)},$$

The normalised stiffness (*K*_n_) for the *n*–*m* cycle range was calculated based on the following equation:

(2) $$K_{n}\left(n-m\right)=\frac{K\left(n-m\right)}{K_{0}\left(n_{0}\right)},$$

*K*_0_(*n*_0_) represents the stiffness assumed as the initial stiffness and calculated for cycle *n*_0_. The calculation assumes *n*_0_ = 500 cycles, representing the number of cycles required for the load response of the specimen to reach a stable state. The initial value of normalised stiffness equals 1, indicating that the specimen is undamaged. Naturally, this constitutes an approximation, since the calculations begin at a specific number of cycles rather than at the time of starting the machine. If material degradation occurs, the normalised stiffness value will decrease. A value of *K*_n_ = 0 (which is practically impossible to achieve) corresponds to the complete failure of the specimen. A change in the slope of the normalised stiffness curve can be interpreted as an indication of a change in the rate of fatigue-induced material degradation. In general, if the angle of inclination is greater, then the rate of the damage level is greater. Graphs showing changes in normalised stiffness are presented subsequently in this paper, in the section presenting the research results.

#### 2.2.3. Instrumentation for Testing Using Passive Thermography

**Figure 5:**
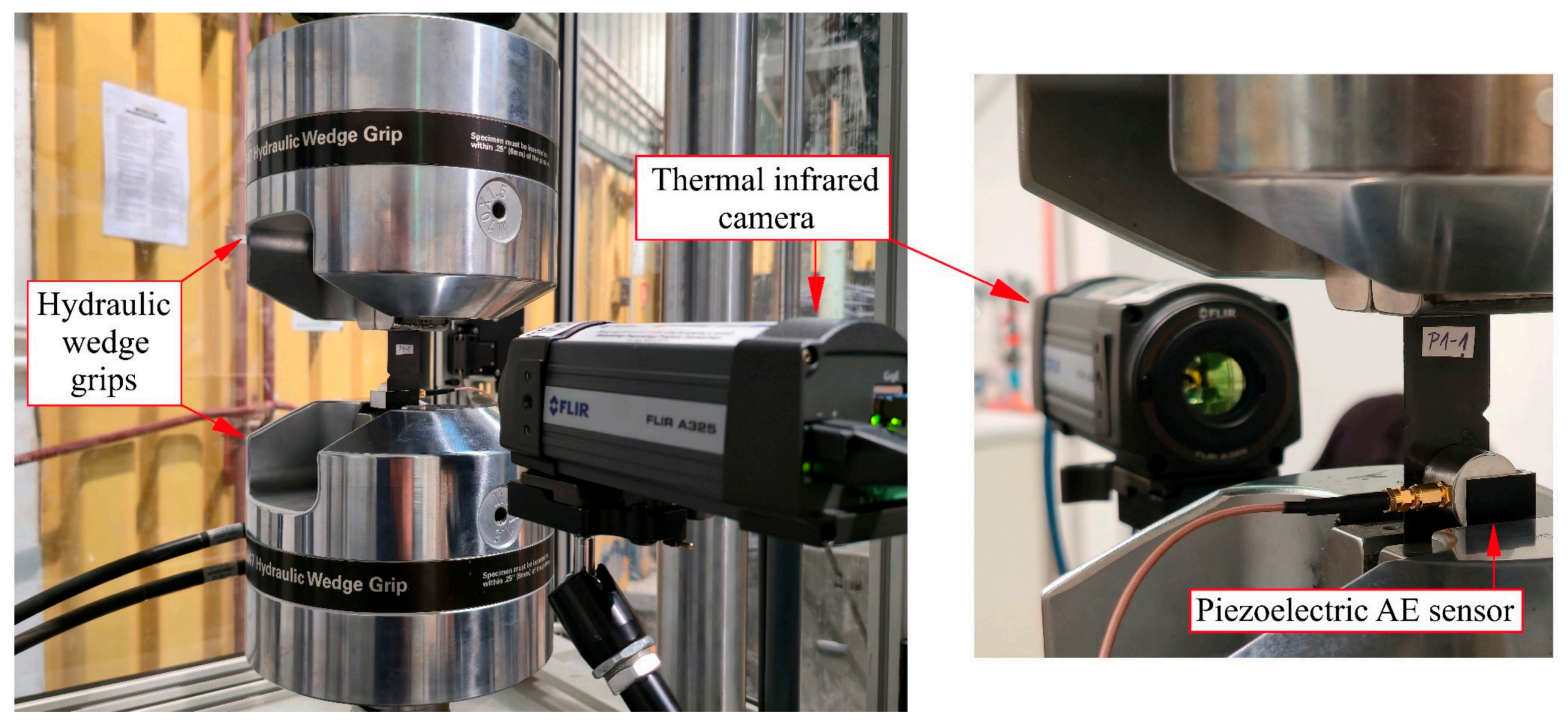
Thermal infrared camera during fatigue testing.

Passive thermography was employed to support research activities assessing thermal changes in test specimens. This equipment allowed for determining the temperature gradient (increase) during fatigue testing and identifying the most damage-susceptible areas. Passive thermography is an imaging method that involves measuring and analysing the natural thermal radiation emitted by an object without heating or stimulating it externally. This non-invasive, non-contact technique allows thermal images to be created based on recorded thermal energy. This study used passive thermography to assess the impact of an increase in temperature on composite specimens undergoing fatigue testing. For the signal recorded using passive thermography, the temperature gradient (Δ*T* difference between maximum *T*_max_ and reference *T*_ref_ temperatures) was expressed by the following equation:

(3) ∆*T* = *T*_max_ − *T*_ref_,

Thermography tests were performed using specialised equipment, including an FLIR thermal infrared A325 camera (FLIR Systems, Inc., Wilsonville, OR, USA)—Figure 5 [38,53]. The analysis was performed using an FLIR A325 thermal imaging camera and the following software: IrNDT v1.7.2 as well as ThermaCAM Researcher Pro 2.10. The camera’s technical specifications were as follows: resolution: 320 × 240 pixels; frame rate: 60 Hz; spectral range: 7.5–13 μm; and temperature range: −20 to +350 °C. The thermal response of the specimens was monitored throughout the heating and subsequent cooling phases.

#### 2.2.4. Instrumentation for Testing Using Acoustic Emission and Description of Investigated Parameters

**Figure 6:**
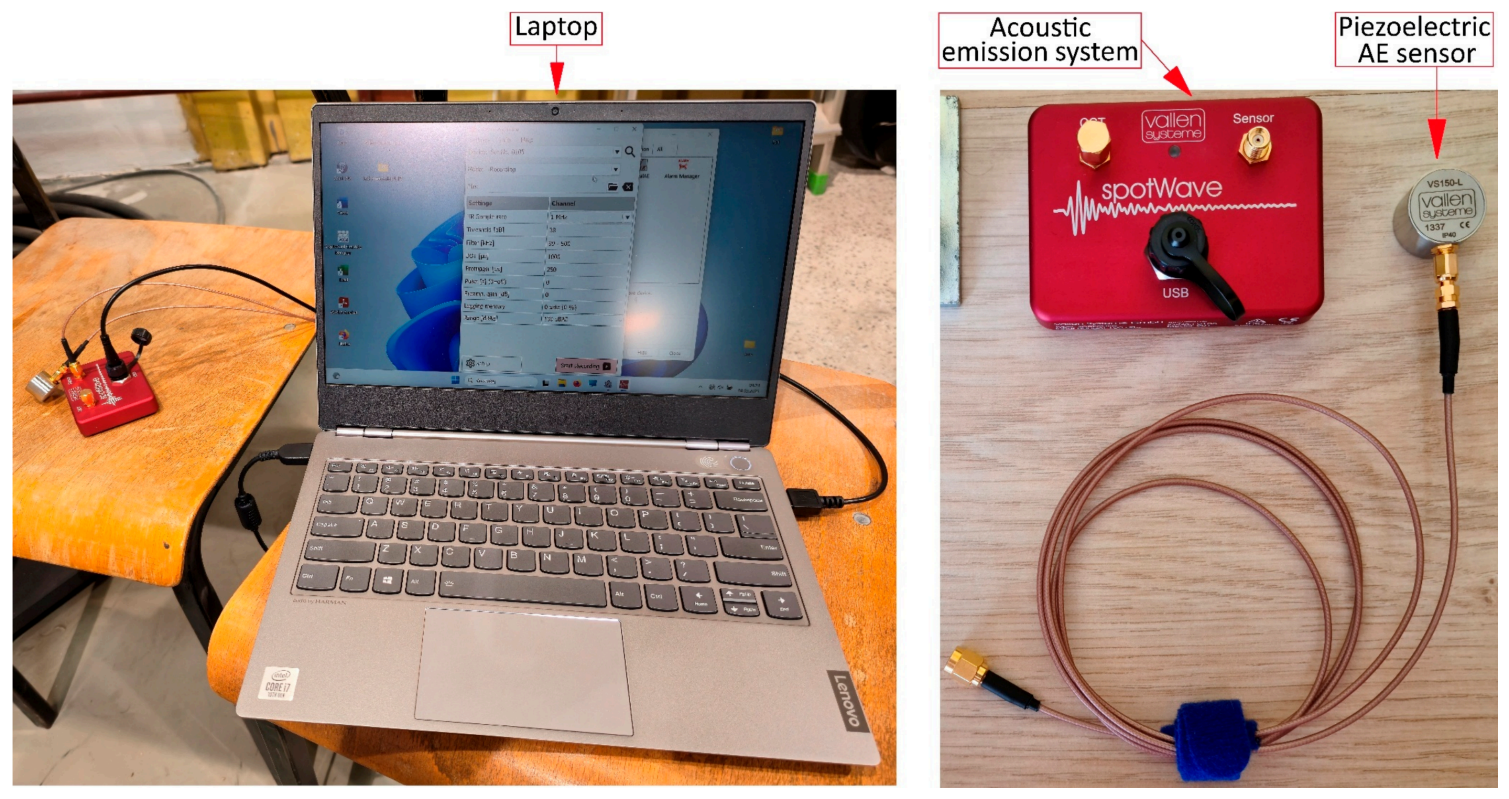
A single-channel system for testing acoustic emissions.

For tests based on acoustic emission (AE), specialised equipment was used to enable the recording of several independent acoustic signals. The SpotWave 201 device (Vallen Systeme GmbH, Wolfratshausen, Germany) used in the research is a portable, single-channel acoustic emission measurement system [54]. It is a fully functional acoustic emission (AE) measurement system comprising: detection, signal conditioning, signal measurement, analysis and output of results. The built-in operating system facilitates the recording and processing of acoustic emission data and the acquisition of transient waveforms. Measurement data is stored in the host device in a *.pridb and *.tradb file. The operating parameters of the AE system are as follows: sampling rate (2 MHz, 16-bit)—freely selectable decimation factor; input range (±100 mV, 100 dB_AE_); analogue filters (highpass: 20 kHz, 2nd order, lowpass: 500 kHz, 6th order); digital filters (Butterworth IIR cascaded biquads)—freely selectable frequencies and filter order; temperature range (−20 to +60 °C); weight (107 g). All test parameters were recorded using specialised spotWave Acquisition (R2022.0809.3) software. The software—spotWave Acquisition is part of the Vallen AE Suite software (R2022.0809.3). The recorded results were analysed using the VisualAE software—Vallen Control Panel (R2022.0809.3) (it is possible to perform analyses on the basis of wavelet transform—WT). The research was conducted at a sampling rate of 1 MHz. All measurements were conducted using a specialised piezoelectric sensor VS150-L passive piezoelectric AE sensor(Vallen Systeme GmbH, Wolfratshausen, Germany) designed primarily for testing composite materials. The VS150-L sensor’s frequency response has a peak at 150 kHz (where it exhibits resonance). This sensor is dedicated to inspecting objects that have no ferromagnetic surface, such as composites and aluminium alloys. The operating parameters of the piezoelectric sensor are as follows: frequency range (100–450 kHz); temperature (−50 to +100 °C). A single-channel AE testing system is demonstrated in Figure 6.

Specific acoustic emission signals were registered during testing, enabling further interpretation of the results. The amplitude *A* of the signal recorded during the test trials is calculated according to the following formula:

(4) *A* = 20 ∙ log(*ALIN*).

The parameter *A* represents the peak amplitude of the acoustic emission burst signal in units of dB_AE_ (derived from the measured peak amplitude in µV). *ALIN* denotes the linear peak amplitude of the burst signal, whose resolution depends on the input range of the measurement (channel). The amplitude resolution is approximately 3.05 µV, for an input range of 100 dB_AE_ (100 mV). From the perspective of conventional AE testing, the signal energy is one of the key parameters for characterising the recorded events. The energy signal *E* was calculated based on the following formula:

(5) $$E=∆t\sum_{i=1}^{N}u_{i}^{2}$$

*E* corresponds to the signal energy of the burst signal scaled to the arbitrary unit of eu. The signal energy is the sum of squared voltage samples (*u*_i_) beginning with the sample of the first crossing of the threshold (*i* = 1) up until the sample with which the duration discrimination time (DDT) expired (*i* = *N*). Variable Δ*t* refers to the time difference occurring between two sequential voltage samples. From a mathematical standpoint, the conversion to a true energy *TE* is done the following way:

(6) $$\mathrm{TE}=\gamma \frac{1}{R}E$$

Parameter *γ* corresponds to the conversion unit factor of eu to J and *R* to an ohmic resistance, based on the following calculations:

(7) $$\gamma =10^{-14}\frac{V^{2}s}{\mathrm{eu}},R=10\;k\Omega$$

The ohmic resistance is dependent on the input impedance of the device. As the input impedance changes, the measured energy (true) value also changes. Since the division by the resistance is a scaling factor, it is recommended to use arbitrary energy units and convert them to engineering units. During the research activities, the recorded signals represented energy, amplitude, and number of counts. The energy signal was the main signal that was analysed in depth.

#### 2.2.5. Instrumentation for Microscopy Observations

**Figure 7:**
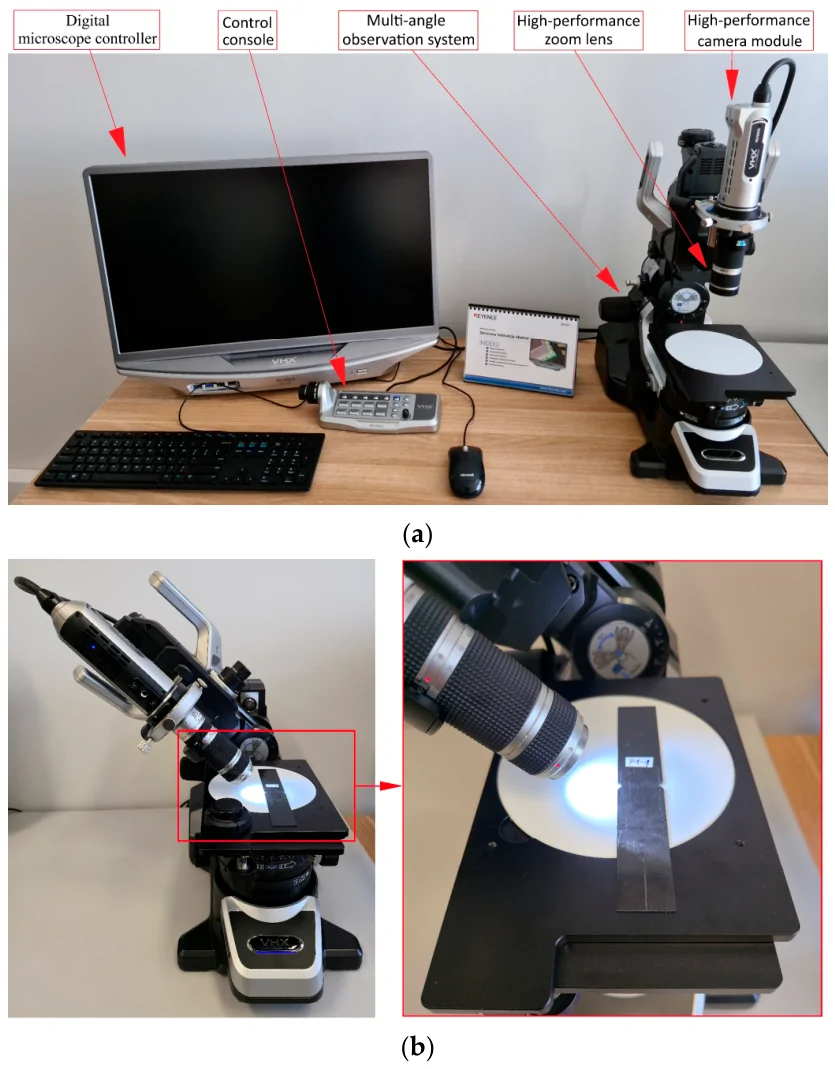
Equipment for digital microscopy testing: (**a**) digital microscope with all necessary accessories; (**b**) observation of a sample test specimen.

Digital microscopy was the last non-destructive testing method used for experimental research. Digital microscopy has primarily enabled the qualitative assessment of the most damaged areas of composite structures during fatigue. A digital microscope controller VHX-970F (Keyence, Osaka, Japan) was used to observe the specimens [54,55,56]. The research was performed using the following microscope components: VH-Z20T lens with a magnification range of 20—200x, VHX-A97FP control console, VHX-7020 high-performance camera module, VHX-S600E multi-angle observation system. All observations during the microscopic examinations were made using 20× magnification. The magnification used was sufficient to assess the forms of damage to structures. The depth-of-field function was employed to improve the visualisation and characterisation of damage in the specimens, with manual focusing of damaged areas located at different distances from the microscope lens. The VH-Z20T lens used for testing features excellent telecentricity and provides extremely clear images, even in deep compositions or 3D images. The aforementioned telecentricity is a property of an optical system in which light rays emanating from it are mutually parallel (the ability to maintain the principal rays parallel to the optical axis in the object space, the image space, or both). Telecentric lenses maintain high image quality and measurement accuracy, regardless of the object’s position. This is crucial for measuring systems, such as microscopes, as well as for generating 3D images and combining information from regions that are out of focus. In addition to the traditional two-dimensional images with depth of field (depth composition), the study used a module to generate three-dimensional visualisations of selected sections of the examined structures. The equipment used for microscopic examination is demonstrated in Figure 7.

All test specimens were subjected to experimental tests using all of the above methods (complementary experimental tests), which were performed in the same procedure. The present study was conducted under a single cyclic tensile-loading condition (10 Hz, 10,000 cycles), with the primary objective of comparing damage evolution in CFRP laminates with different stacking sequences using AE and thermal monitoring. Consequently, the findings are limited to the investigated loading conditions and do not establish general fatigue-life relationships. Future work will investigate multiple stress levels and stress ratios, as well as different loading modes and high-cycle fatigue regimes. This expanded experimental program will enable the development of fatigue-life relationships and a more comprehensive evaluation of the sensitivity and applicability of AE and thermal monitoring for damage characterisation under different fatigue-loading conditions.

## 3. Results

### 3.1. Stiffness Degradation Analysis

**Figure 8:**
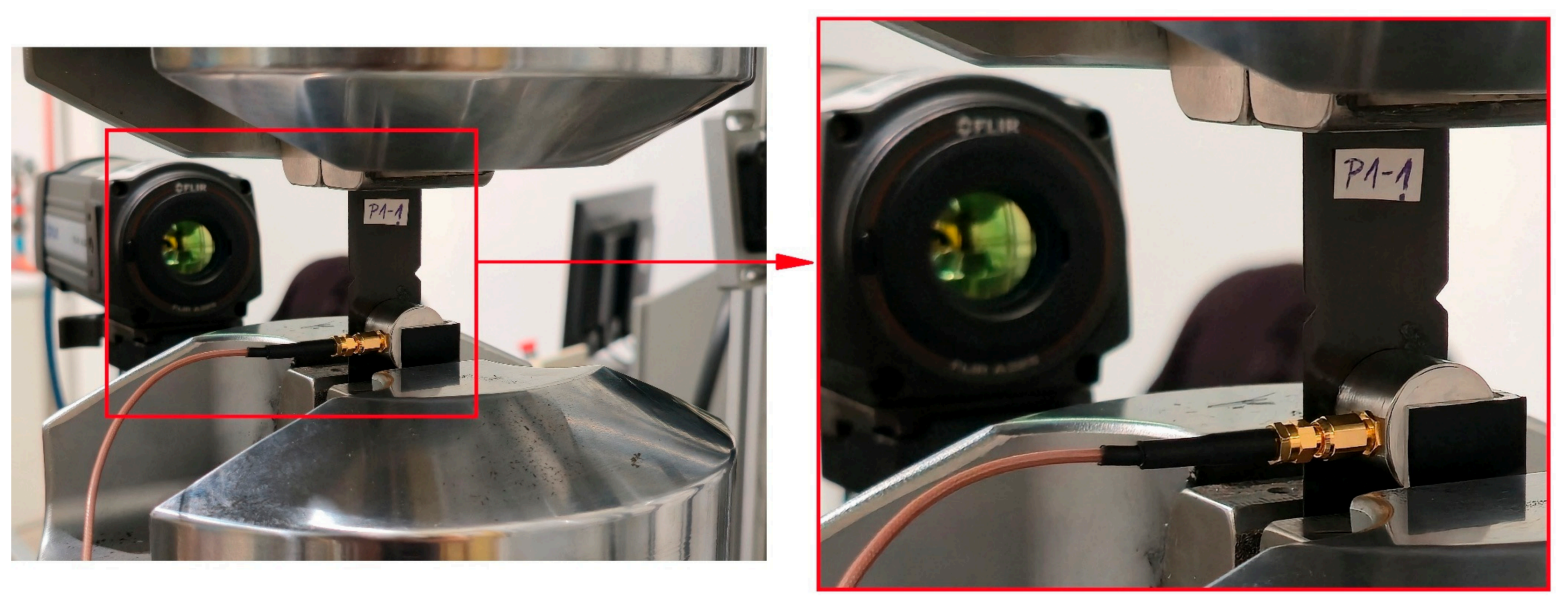
An example test specimen (P1-1) during fatigue testing.

As part of the scientific research, the fatigue response of specimens with different composite layer arrangements (lay-ups) was assessed. For this purpose, complementary experimental testing methods were employed. Figure 8 presents the CFRP profile during testing.

**Figure 9:**
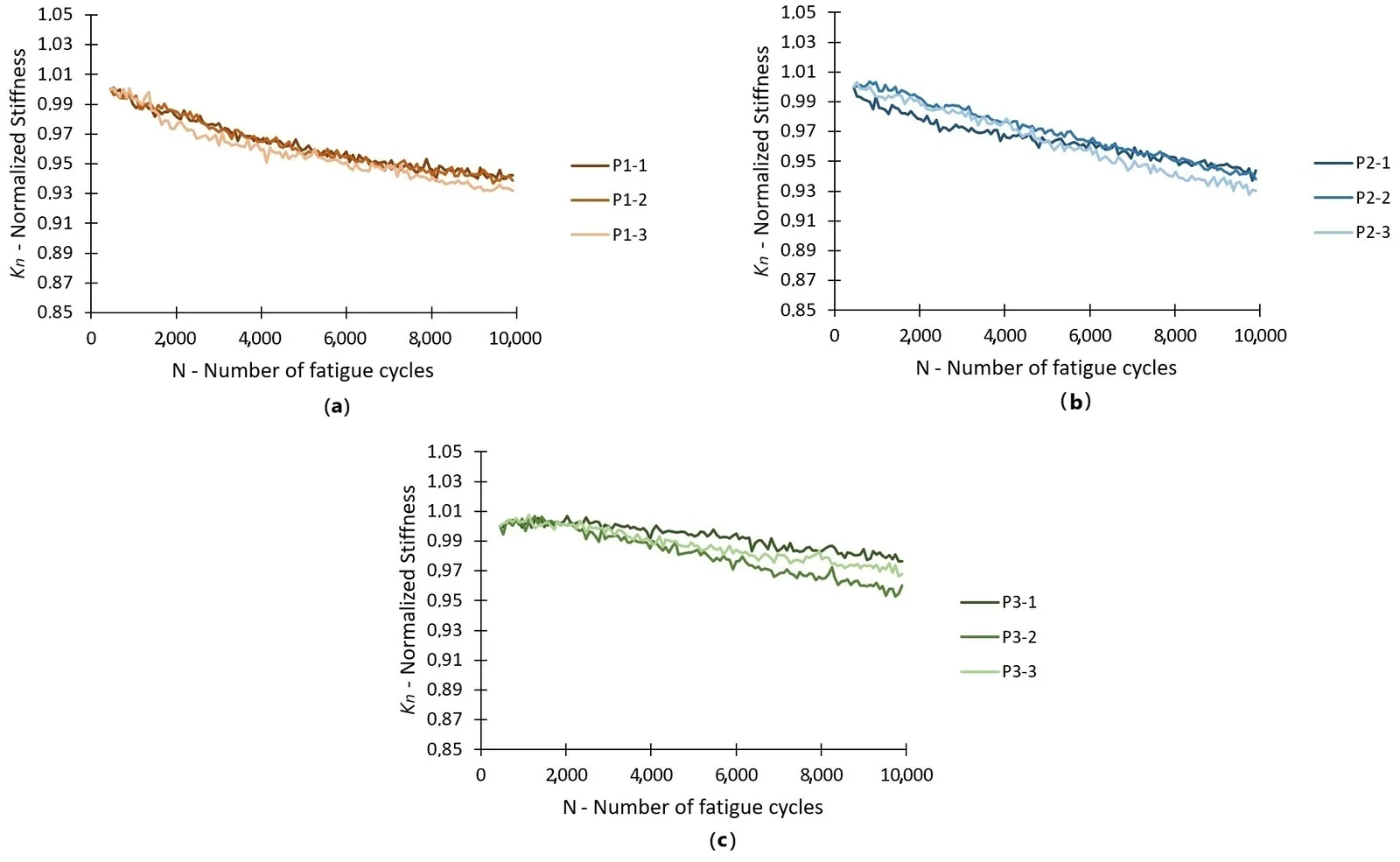
Variation in normalised stiffness as a function of the number of fatigue cycles: (**a**) P1-type specimens; (**b**) P2-type specimens; (**c**) P3-type specimens.

A preliminary assessment of the research results was conducted using results from the fatigue machine. As discussed in detail in the description of the research methodology, the issue of equivalent stiffness of specimens was initially analysed during testing. The study assessed changes in specimen stiffness. The quantitative results of stiffness degradation were presented as changes in the normalised stiffness of the specimens as a function of the number of fatigue (load) cycles. Based on the characteristics of stiffness change, it was possible to assess the impact of the composite lay-up on stiffness degradation. Figure 9 illustrates the variation in normalised stiffness as a function of the number of fatigue cycles.

Analysing stiffness changes allowed for assessing which specimens were more or less susceptible to stiffness reduction during the fatigue study. The average value of the percentage decrease in stiffness was estimated at 6.42% for P1 specimens, 6.58% for P2 specimens, and 3.48% for P3 specimens. Similar results were observed in terms of the decrease in normalised stiffness as a function of the number of fatigue cycles for specimens featuring P1 [0°/45°/−45°/90°]_s_ and P2 [45°/−45°/90°/0°]_s_ lay-ups. Specimens with a P3 [90°/−45°/45°/0°]_s_ lay-up showed the lowest average percentage reduction in normalised stiffness, demonstrating the highest fatigue stiffness. Stiffness analysis of the specimens showed an influence of layup on the change in the fatigue stiffness of composite structures. The specimens demonstrated a relatively linear reduction in normalised stiffness.

### 3.2. Passive Thermography Analysis

**Figure 10:**
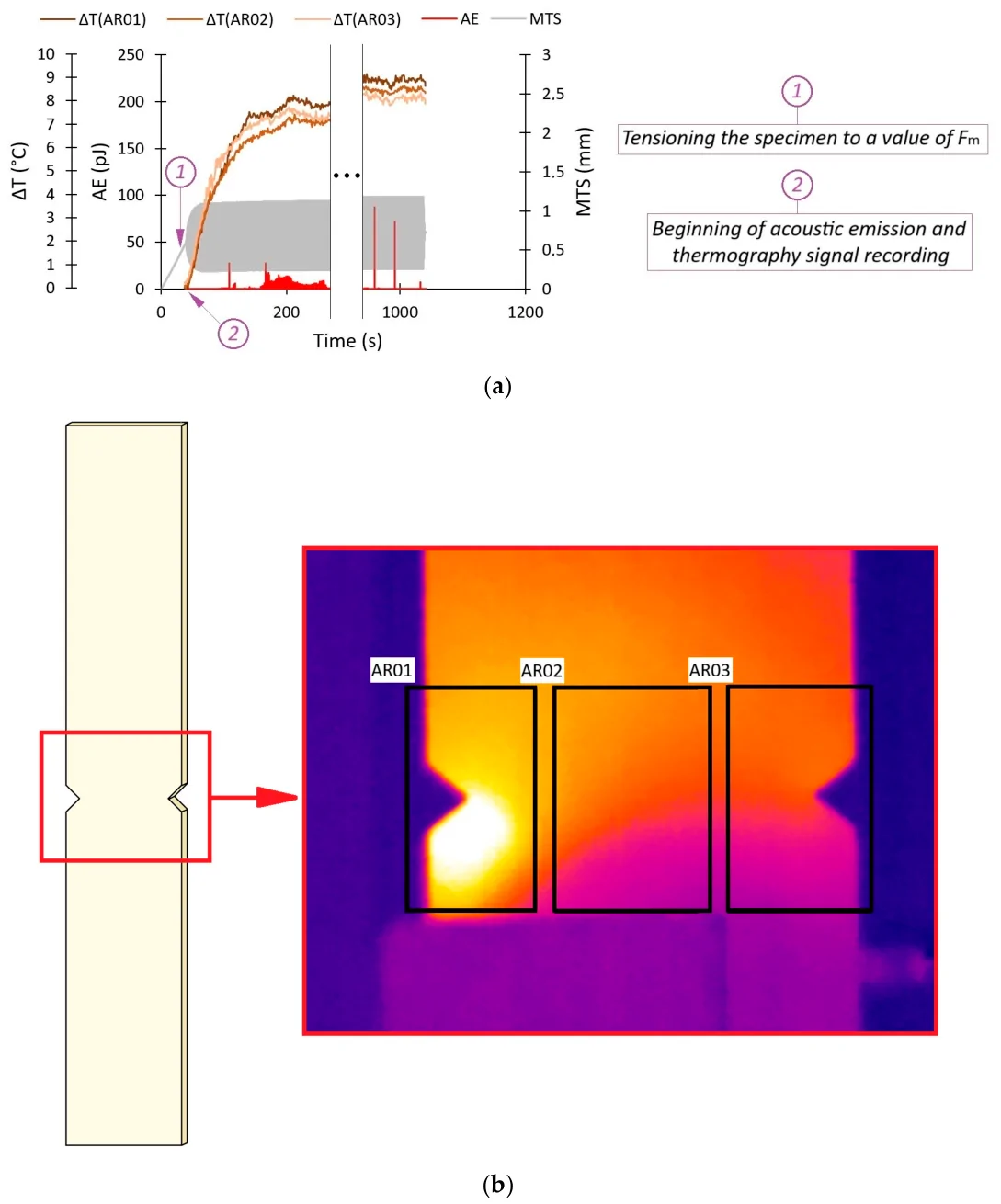
Information on registering test parameters: (**a**) the initial stage and types of signals in experimental research (specimen P1-1); (**b**) areas where a change (an increase) in the temperature parameter was recorded.

An important research objective was to assess the fatigue response of composite specimens using complementary NDT methods. In this regard, using passive thermography and AE in parallel enabled the recorded signals to be assessed independently when evaluating material damage during fatigue testing. In passive thermography, the recorded signal was a temperature gradient (Δ*T* = *T*_max_ − *T*_ref_), while in acoustic emission, it was the acoustic emission energy signal. All parameters were recorded in real time throughout the experimental trials. Based on the research conducted, experimental characteristics were developed, taking into account specimen elongation (marked in grey), temperature (gradient) increase (marked in different shades of brown—P1-type, blue—P2-type, and green—P3-type) and AE energy signal (marked in red). All parameters were presented in relation to a common argument: time. Figure 10a shows part of an example characteristic (specimen P1-1) and explains the initial phase of experimental testing. The chart presented in the figure below (Figure 10a) has been simplified in order to better visualise issues that are relevant from a research perspective. Furthermore, the chart (Figure 10a) shows three curves of the temperature gradient (increase) parameter corresponding to areas AR01 (left notch), AR02 (between notches) and AR03 (right notch)—Figure 10b.

**Figure 11:**
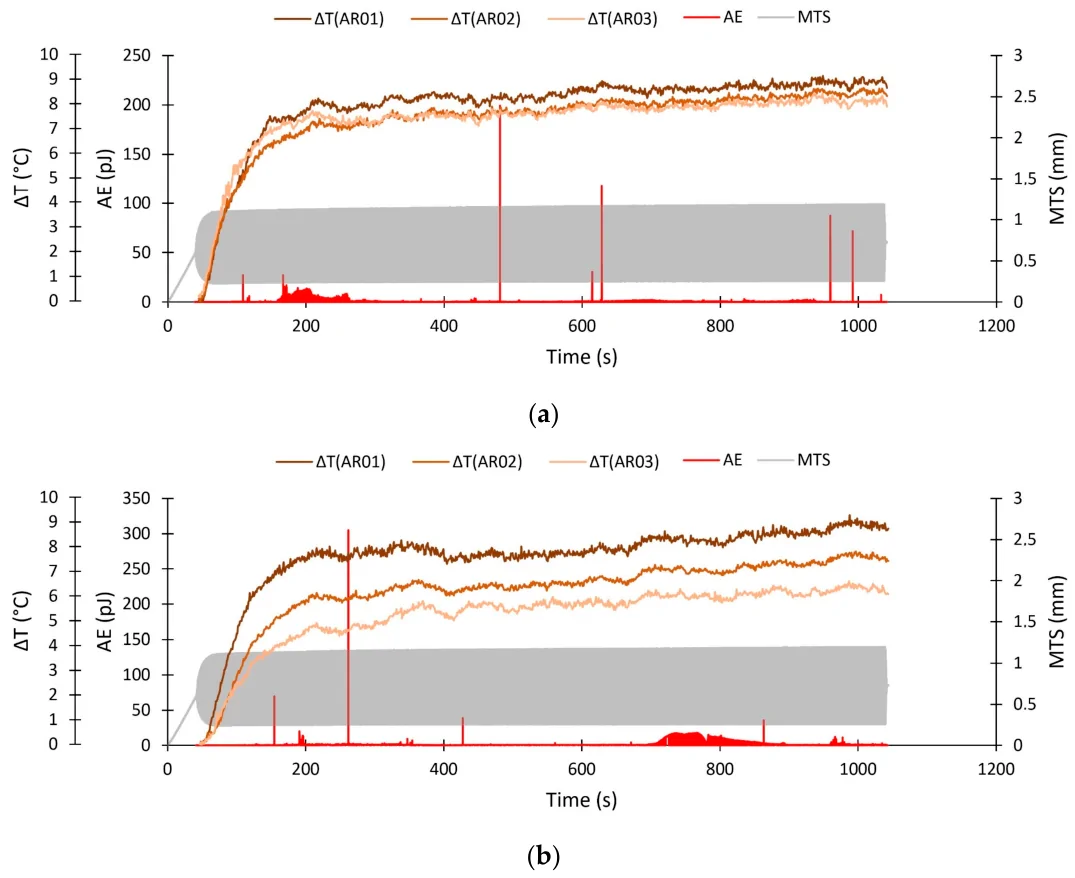
Experimental curves of ΔT, AE, and composite elongation: (**a**) P1-1; (**b**) P1-3.

**Figure 12:**
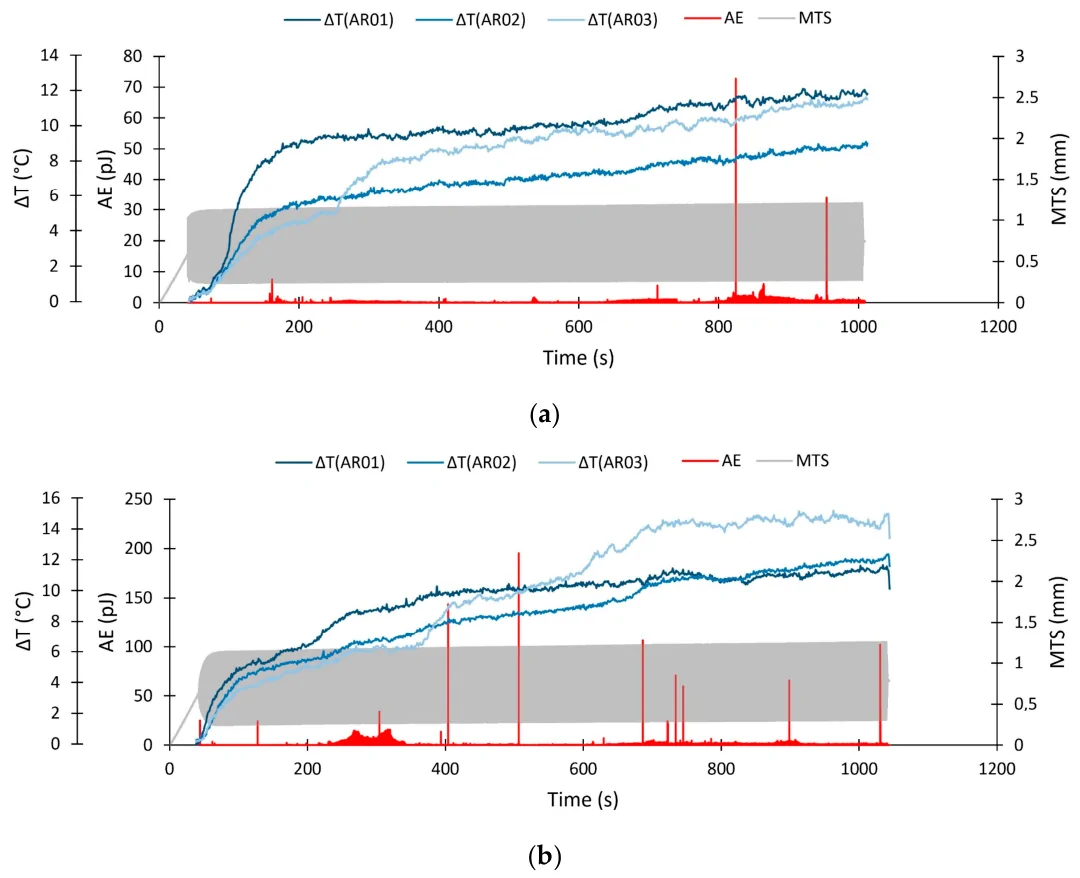

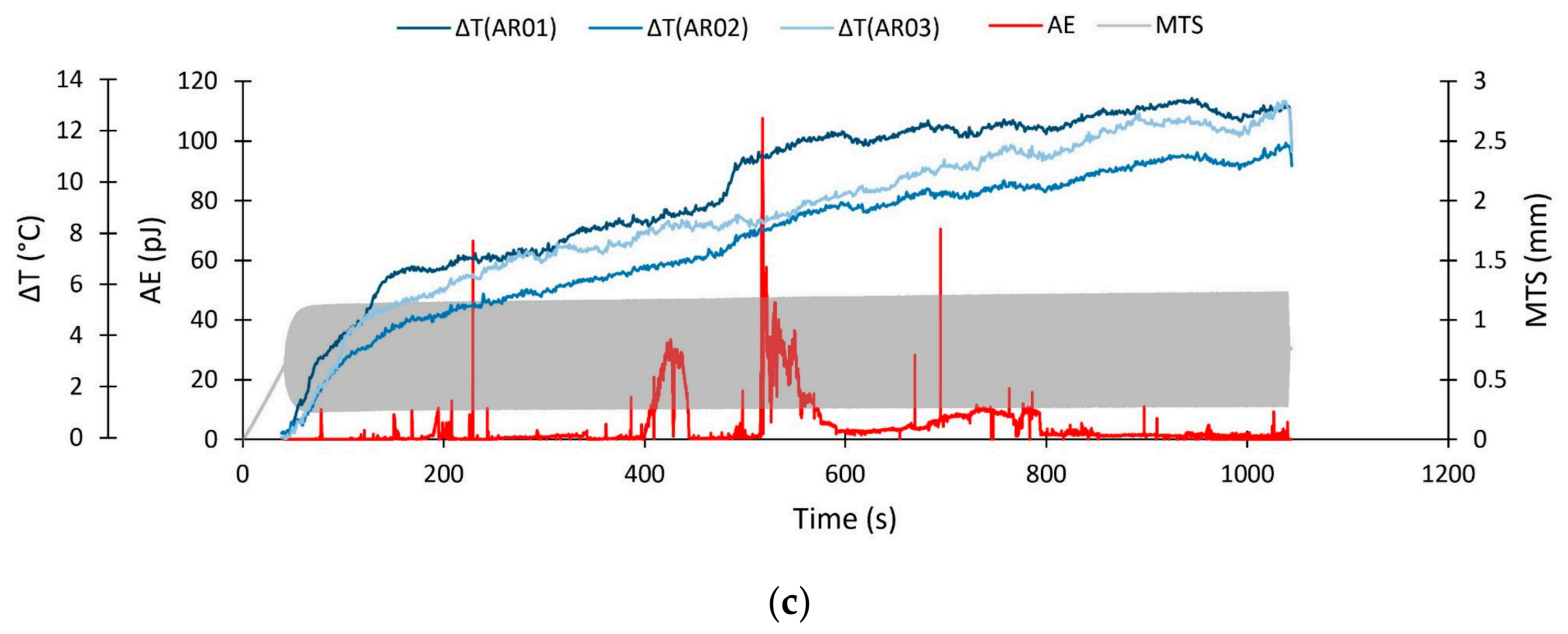
Experimental curves of ΔT, AE, and composite elongation: (**a**) P2-1; (**b**) P2-2; (**c**) P2-3.

**Figure 13:**
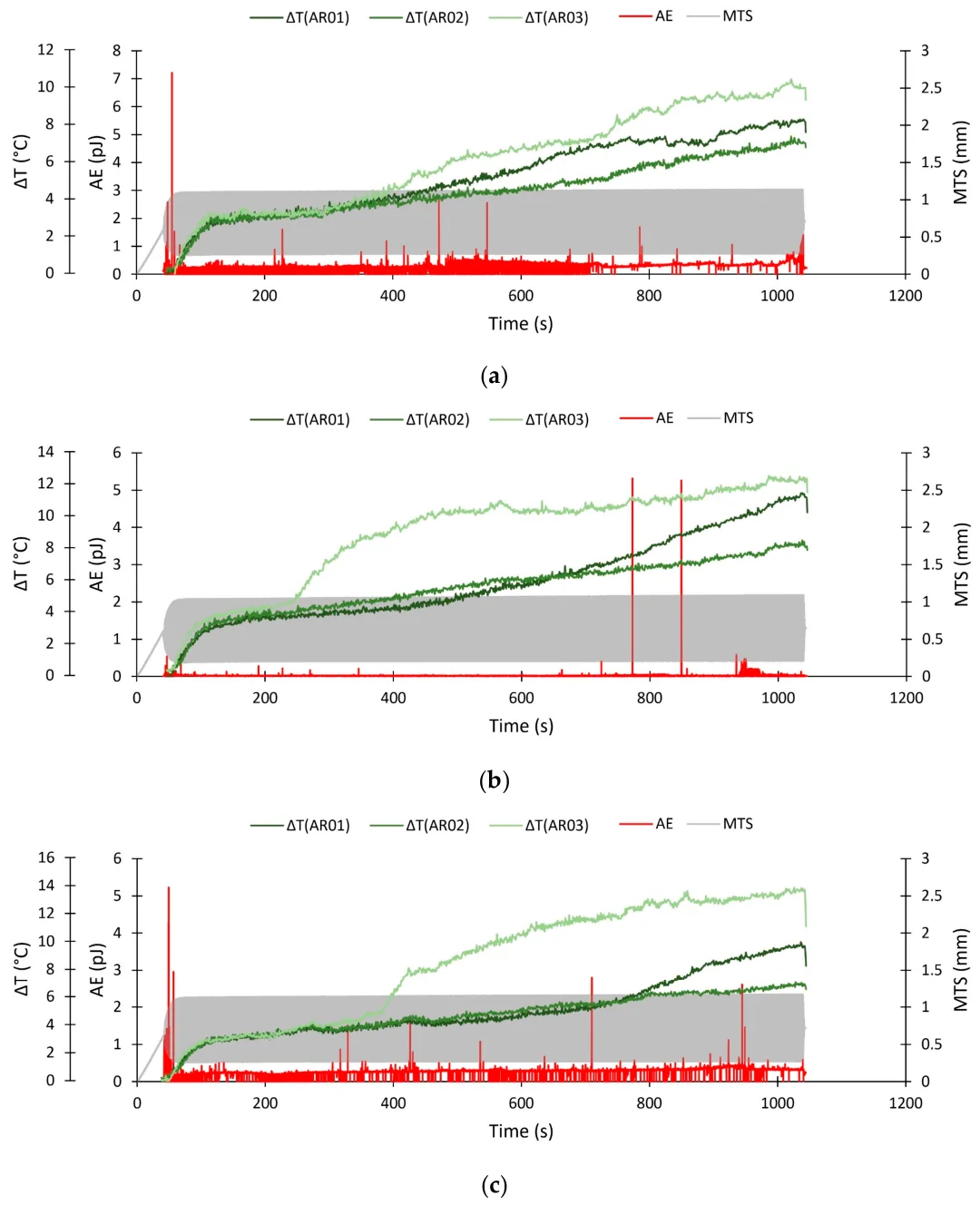
Experimental curves of ΔT, AE, and composite elongation: (**a**) P3-1; (**b**) P3-2; (**c**) P3-3.

The parameters shown in the legends of Figure 10 are as follows: ΔT—temperature (change) increase (°C), AE—acoustic emission energy signal (pJ), MTS—specimen elongation (mm). The first stage of the tests involved tensioning the specimens to an average fatigue load (*F*_m_). The fatigue testing stage then began, during which acoustic emission and passive thermography parameters were recorded. All specimens were subjected to fatigue tests in the same manner. During the experimental tests, a problem occurred with the parameters recorded for specimen P1-2 (both in terms of temperature increase and AE energy signal); therefore, this one characteristic was omitted. All test parameters were recorded for all other specimens, except for the one case mentioned above. Figure 11, Figure 12 and Figure 13 show the characteristics mentioned above.

### 3.3. Acoustic Emission Analysis

Based on the research conducted, the impact of changes to the specimens resulting from fatigue loading was analysed using the recorded test parameters. Initially, the characteristics representing the elongation parameter were analysed using a fatigue machine. Regardless of the type of test specimen, it was observed that an elongation value of approximately 0.6 mm was achieved after just 40 s during the tensioning stage (up to the average load value *F*_m_) of the samples for the standard fatigue tests. During typical fatigue tests, after the initial tensioning of the specimens, the elongation parameter oscillated within a range of approximately 0.2 mm to 1.2 mm. The elongation curves showed negligible differences—all specimens provided extremely comparable curves in terms of quantity and quality. Fatigue testing was performed within the low-cycle fatigue strength range. Therefore, there was no significant increase in elongation during the final stage of the tests (total failure of the specimens was not analysed). The key aspect of the research was assessing damage initiation using complementary research methods to determine the first detectable signs of damage within the composite.

One of the signals analysed was an AE (energy) signal, which was examined in the context of a non-destructive testing method. In the case of acoustic emission signals, a high irregularity in the signal waveform (course) was observed, even in specimens from the same series. Recording signals using AE is highly sensitive to defects in the material’s internal and external structure (e.g., fibre damage, layer fracture and delamination). Some significant peaks in the acoustic emission signal were observed in specimens of the first P1-type, indicating locally occurring material damage. For specimen P1-1, the maximum local signal increase was 199 pJ (481 s). The other significant local signal increases were 118 pJ (628 s), 87 pJ (959 s) and 72 pJ (992 s). For specimen P1-3, the maximum local signal increase was 305 pJ (262 s). The other significant local signal increase was 70 pJ (155 s). For specimen P2-1, the maximum local signal increase was 73 pJ (825 s). The other significant local signal increase was 34 pJ (945 s). For specimen P2-2, the maximum local signal increase was 195 pJ (506 s). The other significant local signal increases were 144 pJ (404 s), 106 pJ (686 s), 71 pJ (734 s), 60 pJ (745 s), 66 pJ (899 s) and 102 pJ (1031 s). For specimen P2-3, the maximum local signal increase was 104 pJ (517 s). The other significant local signal increases were 67 pJ (229 s) and 70 pJ (695 s). For specimen P3-1, the maximum local signal increase was slightly above 7 pJ (55 s). The other significant local signal increases were slightly below 3 pJ (472 and 547 s). For specimen P3-2, the maximum local signal increase was slightly above 5 pJ (773 and 849 s). For specimen P3-3, the maximum local signal increase was slightly above 5 pJ (49 s). The other significant local signal increases were slightly below 3 pJ (57, 710 and 945 s). Any other local signal increases were less significant and have therefore been omitted from the description. For P1-type and P2-type specimens, the local increase in energy signal ranged from several dozen to several hundred pJ, whereas for P3-type specimens, it was only a few pJ. Although maximum energy signal increases were observed at different time points, in the case of specimens P1 and P2, the energy signal showed higher energy peaks than in the case of specimens of type P3. The AE signal course was characterised by high irregularity. All test specimens showed both local increases and concentrations of the energy signal. However, the signal waveform was not clearly repeatable, even for signals recorded in test specimens from the same series. These issues demonstrate the complexity of the damage phenomenon, particularly the initial damage indications observed during the tests, which occurred in each of the specimens.

In the case of the parameter that determines temperature change, it was observed that the maximum temperature increase occurred in the area of the notches. As temperature changes were measured in three independent areas, it was possible to determine precisely which area showed the highest values of this parameter. Based on the results, it was observed that the maximum temperature increase was recorded in the area of the left notch (AR01) for P1-type specimens. The maximum temperature increases were: 9.14 °C (P1-1) and 9.28 °C (P1-3). For P2-type specimens, the maximum temperature increase was recorded at the left (AR01) or right (AR03) notch. The maximum temperature increases were: 12.27 °C (P2-1), 15.17 °C (P2-2), and 13.26 °C (P2-3). Regarding the P3-type specimens, the maximum temperature increase was recorded in the area of the right notch (AR03). The maximum temperature increases were: 10.42 °C (P3-1), 12.51 °C (P3-2), and 13.84 °C (P3-3). The highest temperature increases were recorded for specimens of P2-type and P3-type. The lowest temperature increases were recorded for specimens of P1-type. Maximum temperature changes were not recorded in the area between the notches (AR02) in any of the analysed cases. This suggests that the left or right notches are the areas most susceptible to damage in the context of the recorded temperature changes.

**Figure 14:**
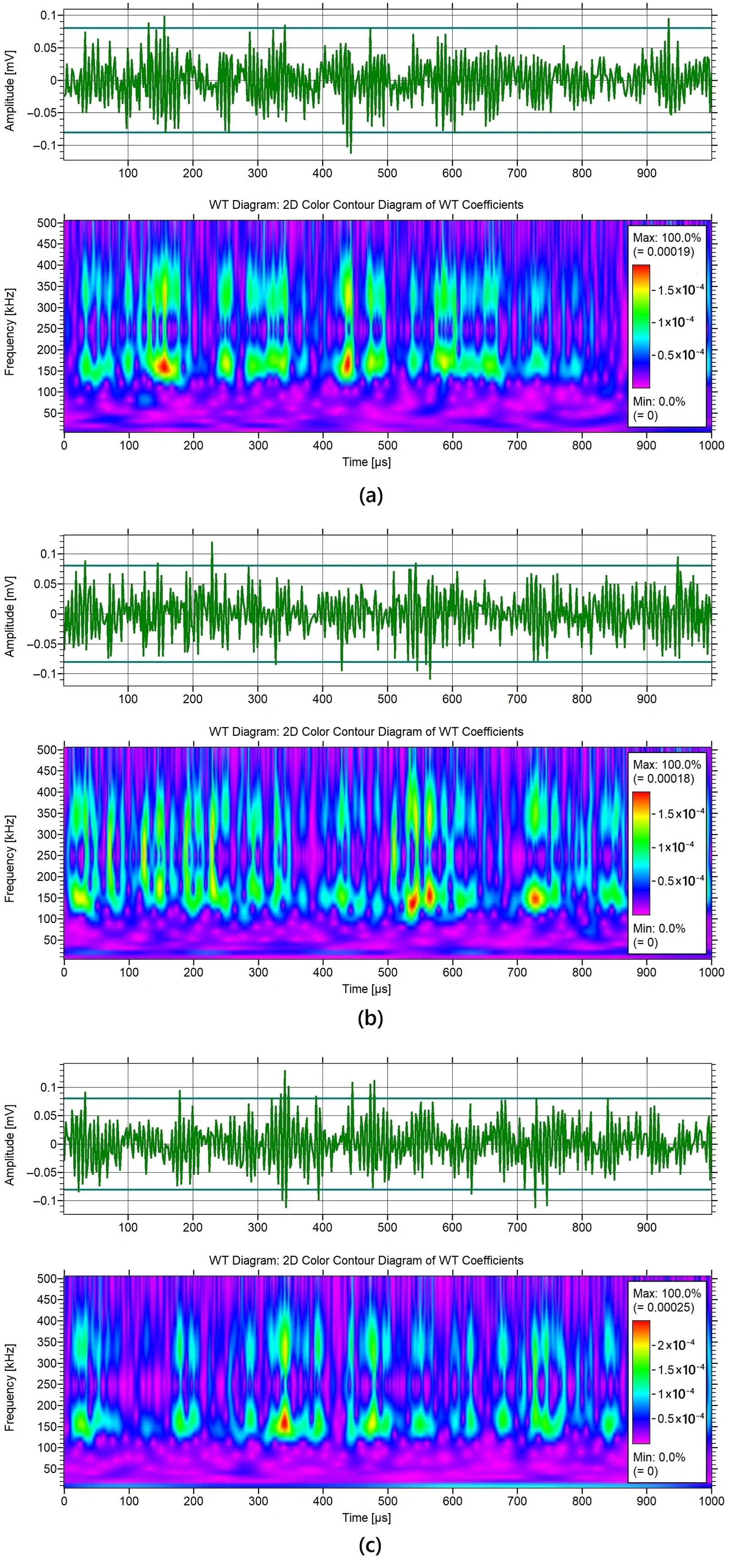
WT contour plot and transient response waveform: (**a**) specimen P1-1; (**b**) specimen P2-1; (**c**) specimen P3-1.

Additionally, preliminary identification of early-stage mechanical damage was performed using an additional testing method based on data obtained from AE (based on the recorded *.tradb file—transient data). Preliminary indications of mechanical damage within the composite material structure were identified through wavelet transform analysis. The wavelet transform (WT) was employed to investigate non-stationary signals whose frequency and amplitude characteristics vary over time. This approach enables the temporal evolution of the signal in the time-frequency domain to be characterised, providing detailed information on changes in both frequency content and amplitude. In general, wavelet transform allows the frequency spectrum of a signal to be analysed as a function of time by decomposing the signal into short-duration waveform components, referred to as wavelets, which serve as basis functions [57,58,59]. The wavelet transform diagram shows the magnitudes of the wavelet transform auto-scaled in rainbow colours. The colour represents the WT coefficient values representing signal energy: purple and blue indicate low energy, green indicates moderate activity, while yellow and red indicate the maximum energy for AE events. Figure 14 shows the sample results obtained using the wavelet transform. Sample results were presented at the first significant energy signal peaks during the fatigue tests. The results were shown over a relatively short period of time.

WT coefficients are used in acoustic emission (AE) analysis to examine signals in the time-frequency domain. Compared with conventional signal-processing techniques, such as the Fast Fourier Transform (FFT), the wavelet transform provides a more comprehensive time-frequency representation of the analysed signal. The resulting wavelet coefficients describe the distribution of signal energy across different frequency bands and time intervals. This enables the identification and differentiation of various damage mechanisms, facilitates the extraction of relevant signal components from background noise, and supports the detection and characterisation of transient phenomena. WT coefficients can be used to identify wave modes, determine arrival times, and classify AE events. Regardless of the failure mechanism (the damage mechanisms for specimens P1, P2 and P3 are described further on in the study—digital microscopy), local maximum energy signals (red colour) were demonstrated at a frequency of approximately 150 kHz. The WT characteristics are presented based on relatively short time intervals. In each of the analysed cases, it can be observed that the WT coefficients on the frequency-time characteristics had a similar course. Despite analysing an extremely short time period, it has been observed that there is an intensity of signs indicating progressive damage from the beginning of the fatigue tests. Similar failure mechanisms were observed regardless of the composite’s lay-ups (each lay-up was a combination of the same layers: 0°, ±45° and 90°). With a resonance frequency of 150 kHz, the sensor can detect signals with the highest sensitivity due to the natural amplification of mechanical waves by the piezoelectric element. This is advantageous in acoustic emission (AE) testing, where weak signals generated by defects such as microcracks in composites need to be identified. Matching the resonance frequency to the frequencies of these signals improves their detectability. The Vallen VS150-L sensor has an official frequency response range (passband) of 100–450 kHz, within which it effectively detects acoustic waves. The lower limit (below 100 kHz) reduces low-frequency mechanical noise, such as friction and ambient vibrations, while the upper limit (above 450 kHz) includes high-frequency waves, which are often strongly attenuated by the material.

In the frequency range below 100 kHz, no signs of damage were evident; in this range, friction effects and minor damage initiation typically occur. In the 100–200 kHz range, significant changes occurred in the graphs, which is usually consistent with the frequency characteristics reported for matrix cracking and/or delamination in similar material compositions. The 250–400 kHz range also showed important changes in the graphs; this is typically in line with the frequency characteristics reported for fibre debonding and/or interface damage. Changes observed in the frequency range above 350–400 kHz most often relate to carbon fibre fracturing. The degradation process usually involves several stages, most commonly matrix cracking, delamination and fibre breakage. A comprehensive description of the methodology and the interpretation of the obtained characteristics is provided in [59].

In wavelet analyses of composite damage signals, especially those using the acoustic emission (AE) method, there are no universal frequencies that clearly correspond to matrix cracking, delamination, or fibre breakage. However, approximate frequency ranges identified in the literature can serve as a starting point for analysis rather than a rigid criterion for damage identification. Many studies in the literature present the frequencies or frequency ranges at which matrix cracking, delamination, or fibre breakage occurs [60,61,62,63,64,65]. However, there are no strictly defined frequency bands for comparison with results from one’s own research. One consistent finding across the literature is that matrix cracking and the beginnings of delamination occur first at low frequencies, followed by delamination and damage to the fibre-matrix interface at medium frequencies, and finally, fibre cracking at higher frequencies. Among the possible failure mechanisms, it is important to note that delamination occurs over the longest period of time in the frequency-time plots.

The AE signals exhibited substantial variability, including among specimens with the same stacking sequence. Therefore, the frequency ranges associated with matrix cracking, delamination, and fibre fracture should be interpreted as indicative features rather than unique signatures of individual damage mechanisms. Signal characteristics may be influenced by wave propagation, sensor response, specimen geometry, and the occurrence of multiple interacting damage processes. Furthermore, the proposed assignments were not independently validated using internal imaging techniques, such as X-ray computed tomography. This limitation should be considered when interpreting the results. Future work will combine AE monitoring with complementary imaging and microscopic examination to establish more reliable correlations between signal characteristics and the underlying damage mechanisms.

### 3.4. Complementary Non-Destructive Observations

A more comprehensive approach would be to analyse three parameters simultaneously: frequency, time of occurrence, and wavelet transform (WT) coefficient energy. Further research and scientific investigations will focus on this approach in the near future.

**Figure 15:**
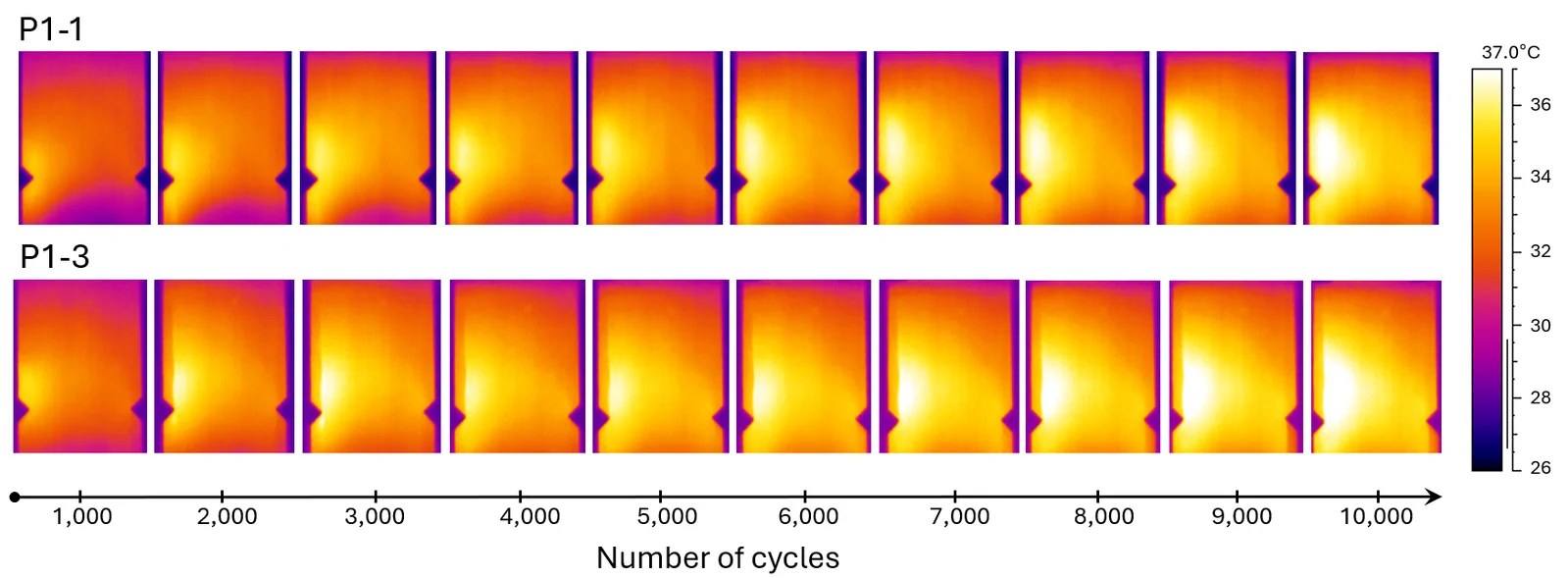
The temperature distribution during the fatigue testing for samples P1-1 and P1-3.

Figure 15 shows the temperature distribution during the fatigue test. In the P1-type specimens, damage to the 0° layer appears early in the fatigue test. This damage causes a significant increase in temperature in the AR01 area (see Figure 11). The initiation of damage on the left side of the specimen determined the further behaviour of the specimen during the test, and a noticeable temperature difference between the left and right sides.

**Figure 16:**
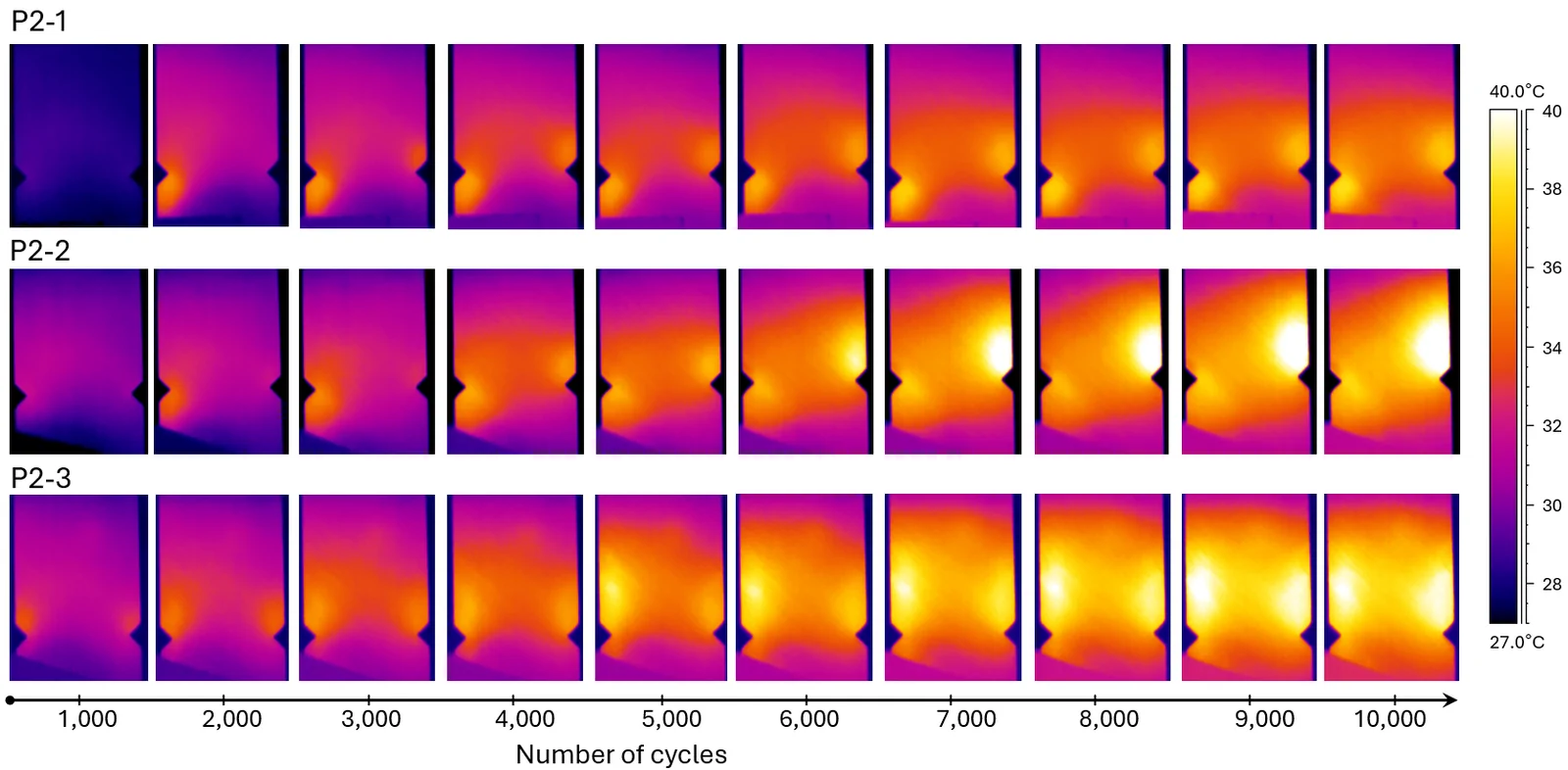
The temperature distribution during the fatigue testing for samples P2-1, P2-2, and P2-3.

Figure 16 presents the progression of the fatigue test based on the surface temperature distribution recorded for the P2-series specimens. The temperature recorded outside the stress concentrators (AR02) enables the progressive degradation of the material, indicated by rising temperatures. Due to the different nature of fatigue damage evolution in this angular configuration, the repeatability of the process, as expressed by the temperature increase, is not precisely maintained. Damage to the 0° layer is less visible in this case because the energy released in the form of a temperature increase, caused by damage to this layer, is dissipated by the ±45° layers. Unlike P1-type specimens, damage can be identified in the form of a sudden temperature increase in a given area over a relatively short period of time. By monitoring these individual areas, we can not only observe material degradation during fatigue but also capture the initiation of local damage and its impact on the overall failure.

**Figure 17:**
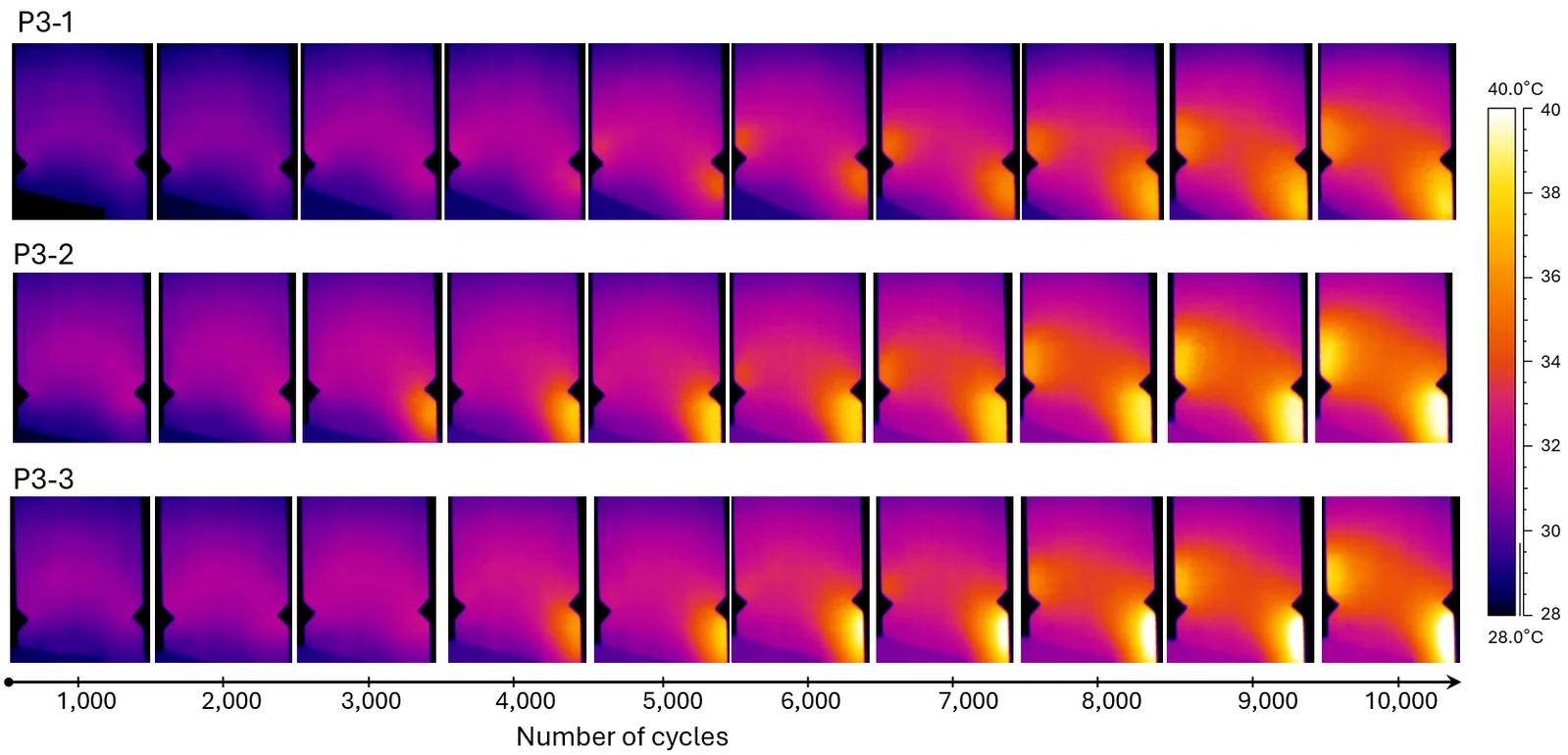
The temperature distribution during the fatigue testing for specimens P3-1, P3-2, and P3-3.

For specimens P3-1, P3-2, and P3-3, the consistency in the initiation as well as evolution of fatigue damage is evident, leading to more uniform temperature increase characteristics, as shown in Figure 17. In specimen P3-1, the fatigue load transfer process is slightly more uniform, resulting in lower intensity and temperature increases. The temperature gradient characteristics in the marked areas clearly indicate that the right side of the specimen is where damage developed for specimens P3-2 (after approximately 2200 cycles) and P3-3 (after approximately 4000 cycles). This damage was characterised by a rapid temperature increase resulting from energy release. No such rapid damage occurred in the case of specimen P3-1, and therefore the temperature increase remained stable.

**Figure 18:**
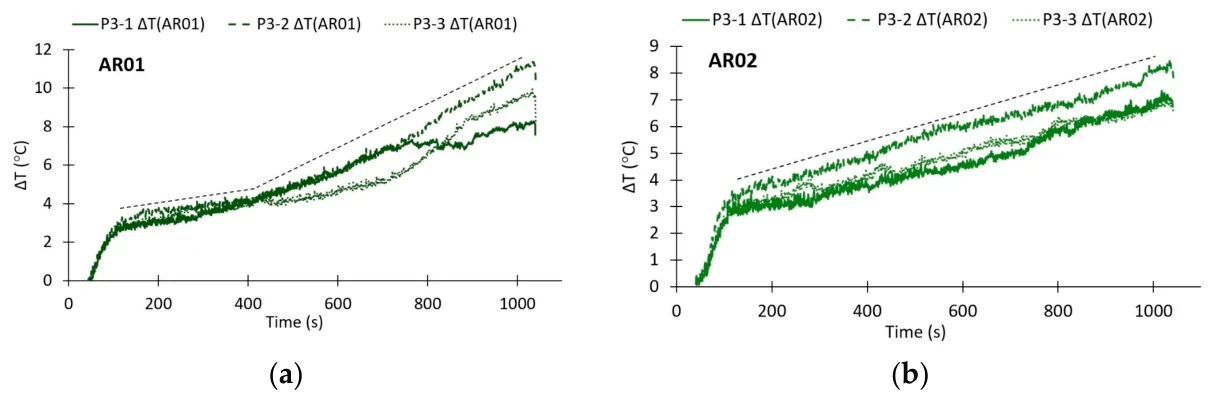
The temperature increase intensity for specimens P3-1, P3-2, and P3-3: (**a**) AR01; (**b**) AR02.

When monitoring the central part of the specimen (outside the stress concentration areas), it is worth noting that the temperature increase intensity is constant (dashed line in Figure 18). This indicates stable, progressive degradation of the material in this area. In the case of area AR01 on the left side of the specimen, however, this intensity changes as a result of sudden damage to the right side of the specimen. The damage below the right notch significantly weakened the specimen, increasing the rate of material degradation. This can be observed in the thermal imaging analysis as an increase in the intensity of the temperature increase.

Analysis of the temperature increase outside the stress concentration zones (AR02) shows that the initial phase of rapid temperature increase after the start of the fatigue test is similar for all specimens analysed. The temperature increase on the surface of specimens P3-type is significantly lower than that in specimens P2-type and P1-type in the second phase of the fatigue test, which shows progressive material degradation. This can be explained by the presence of a 90° layer in specimens P3-type, which acts as a temperature-dissipating barrier. It can be assumed that the 0° layers largely determine the load capacity of the sample. In specimens P2-type, the ±45° layers are located on the outside of the composite structure and are therefore directly monitored by a thermal imaging camera. This results in a significantly higher temperature increase (and thus surface temperature) in this area. Clearly, the specimens from series 2 (P2-type) reach a significantly higher temperature in the initial phase than the specimens from series 3 (P3-type) in the area on the left. In series 3 (P3-type), the ±45° layers are located under the 90° layer. Under the assumed load conditions, this layer does not transfer a significant part of the load. From the perspective of thermal imaging tests, it acts as an intermediate layer in heat transfer. Figures showing temperature distributions indicate that, in series 3 (P3-type), a significant temperature increase is only visible after approximately 3000–4000 cycles, caused by sudden damage at the notch. During the initial phase of the fatigue test for this series, higher temperatures are observed in bands aligned with the ±45° layer orientation, beginning at the specimen’s weakest point (the notches) and spreading to the middle section above the notches. A symmetrical temperature distribution pattern would be expected in the lower part, but this is disturbed in the presented test by the presence of an acoustic emission sensor located below the notches.

All of the results presented above should be interpreted as evidence of the observed experimental trends, rather than as a comprehensive statistical characterisation of the investigated stacking sequences. Due to the small sample size, further statistical analyses were deemed to have limited inferential value and could potentially lead to misleading conclusions. Therefore, no additional statistical tests or error bars have been introduced, and the differences observed among lay-up configurations are discussed cautiously, rather than being interpreted as conclusive evidence of the statistically significant effects of the stacking sequence. Further experiments involving a larger number of specimens are necessary to assess the repeatability of the results and improve the statistical robustness of the comparisons.

### 3.5. Microscopic Observations

**Figure 19:**
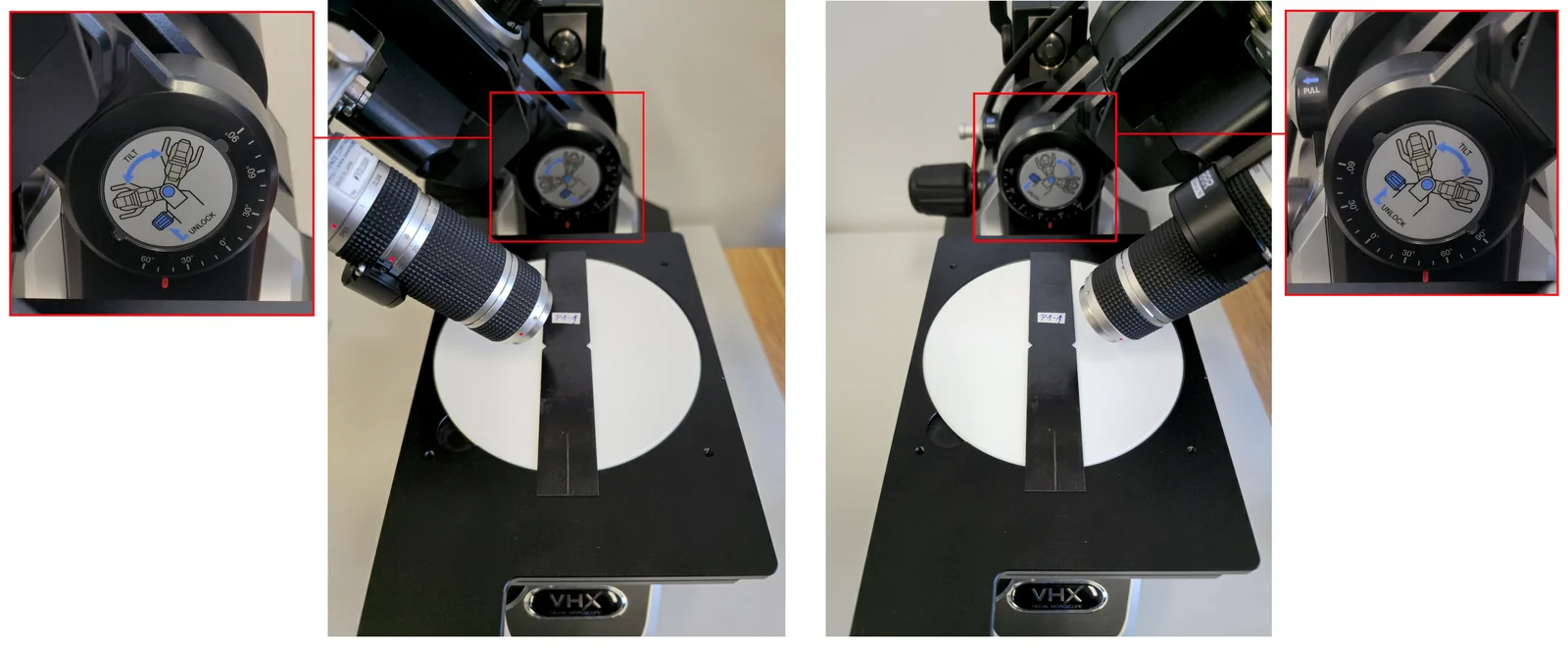
Observation of test specimens and damage occurring during fatigue testing.

An additional phase of the investigation involved a detailed post-fatigue examination of the specimens to identify and document damage within the composite material. The assessment was performed using advanced digital microscopy techniques, which enabled detailed visualisation of the damaged areas. A qualitative evaluation of the damage mechanisms occurring in the composite structures was also conducted. Particular attention was given to identifying the dominant damage modes and determining their precise locations within the specimens. Microscopic observations were performed with the head of the digital microscope (part of the multi-angle observation system) tilted at a 45° angle to the specimens being examined—Figure 19.

**Figure 20:**
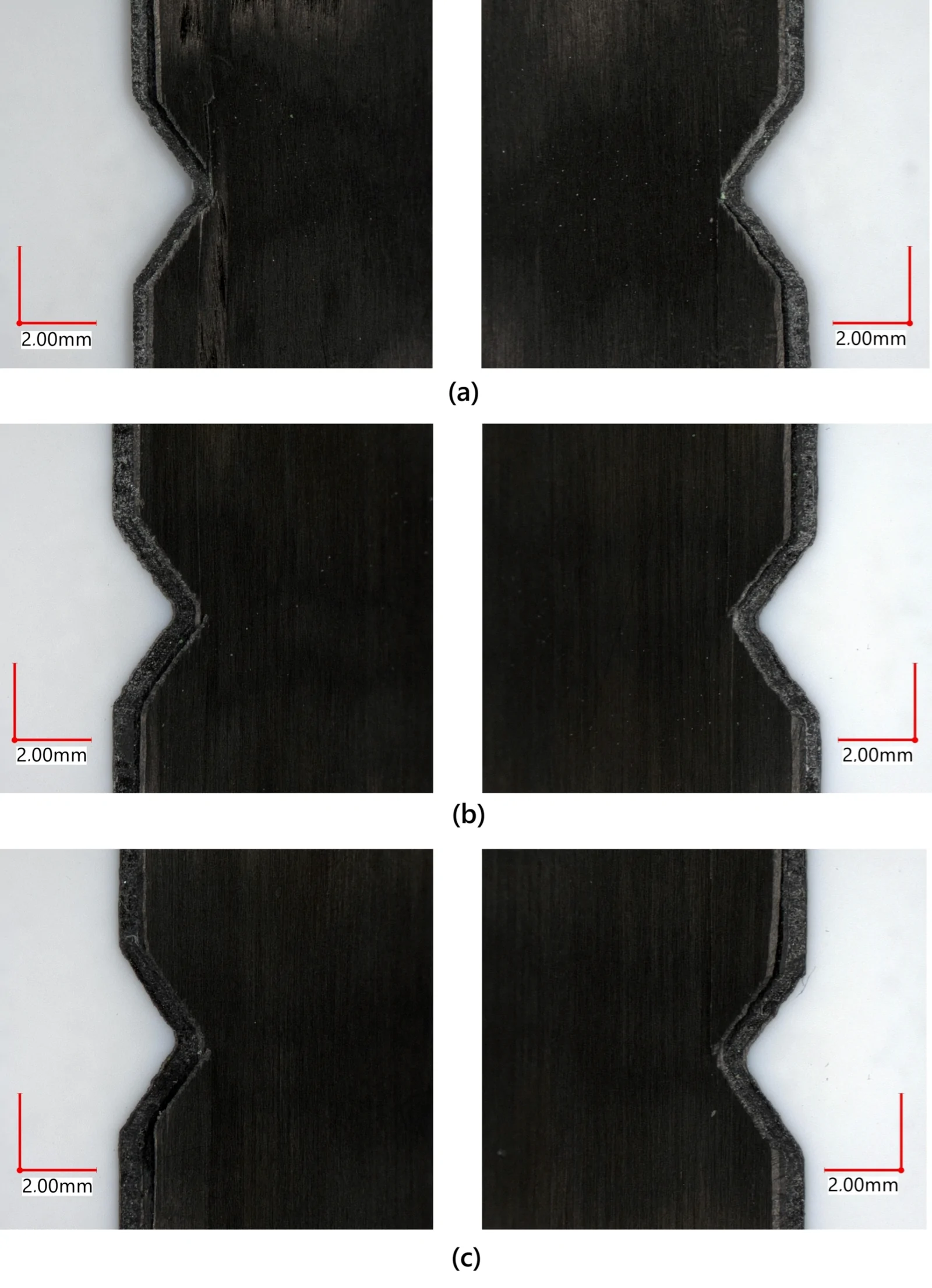
Microscopic assessment of damage: (**a**) specimen P1-1; (**b**) specimen P1-2; (**c**) specimen P1-3.

**Figure 21:**
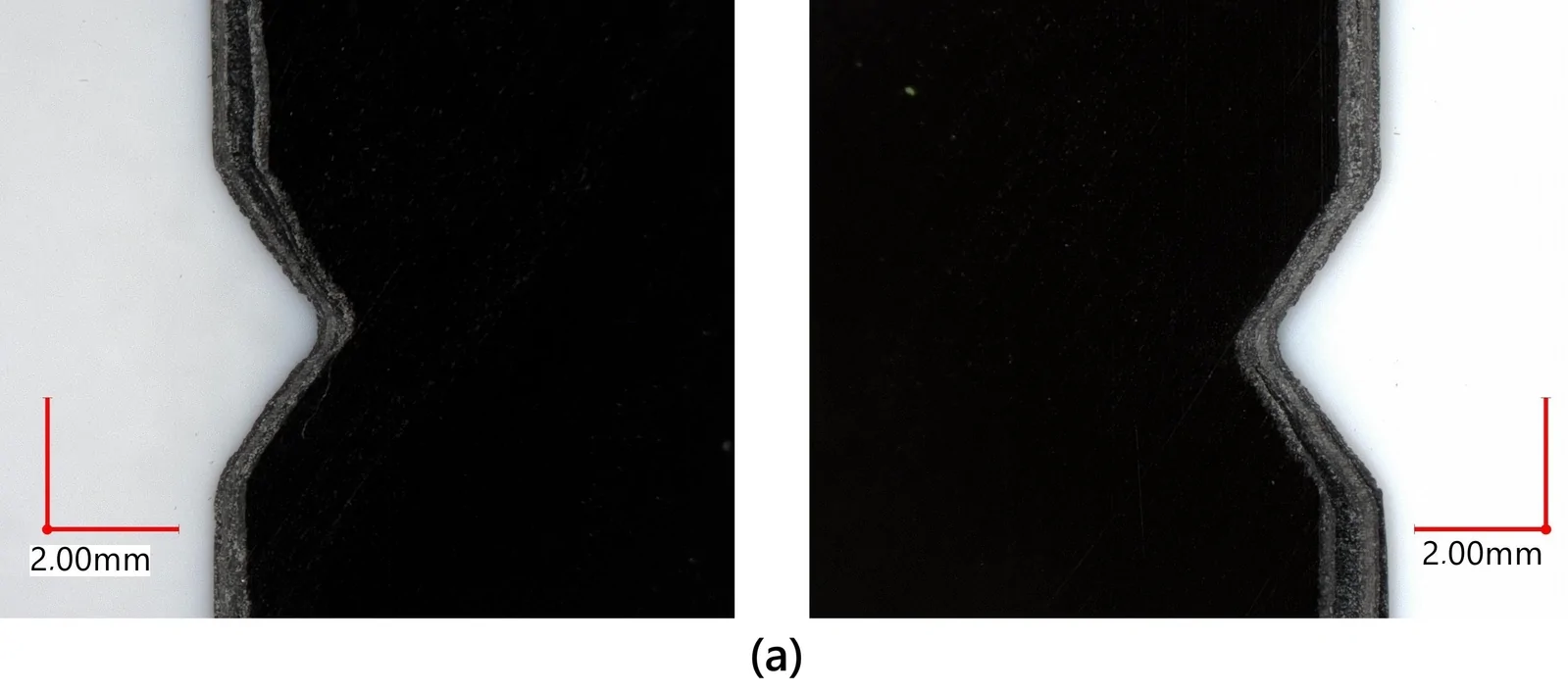

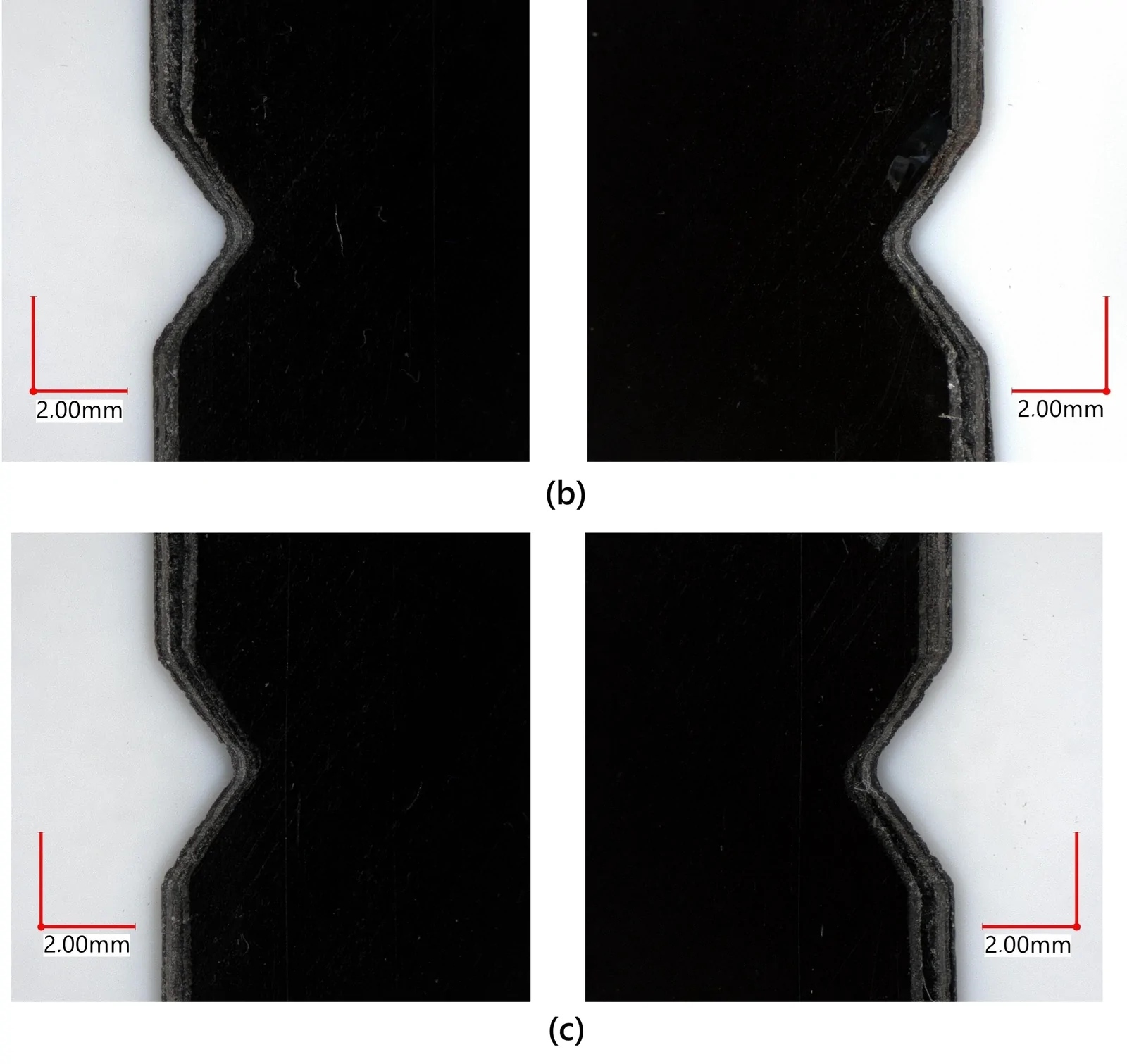
Microscopic assessment of damage: (**a**) specimen P2-1; (**b**) specimen P2-2; (**c**) specimen P2-3.

**Figure 22:**
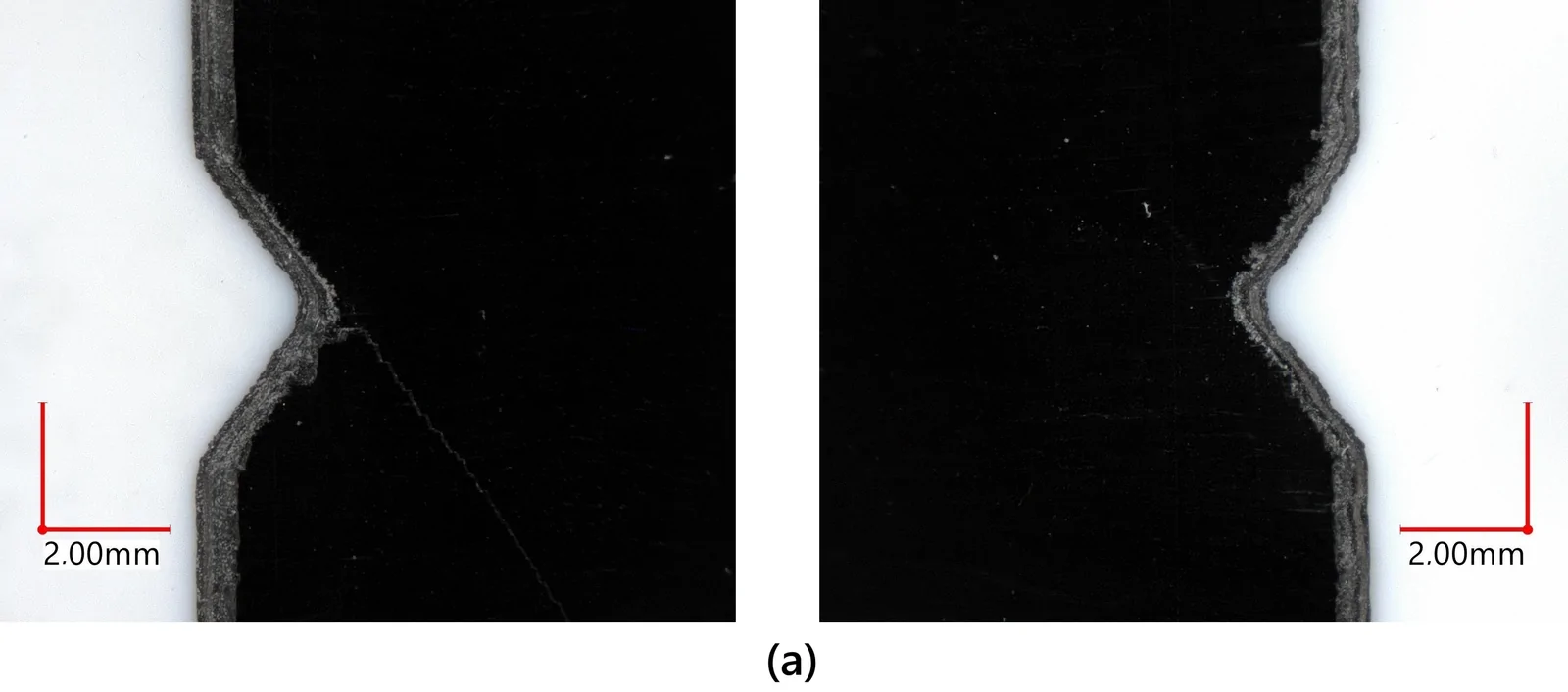

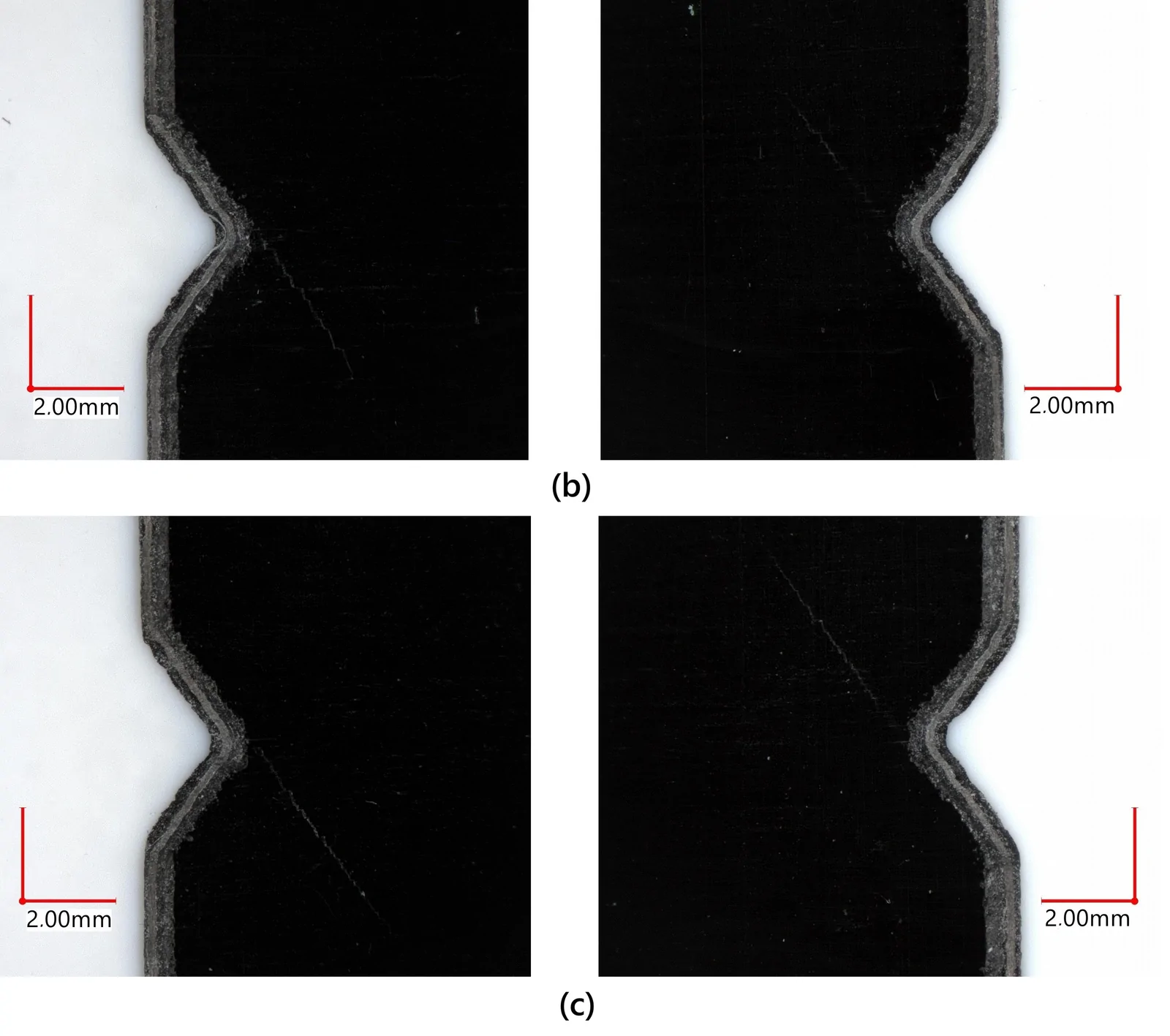
Microscopic assessment of damage: (**a**) specimen P3-1; (**b**) specimen P3-2; (**c**) specimen P3-3.

A special depth-of-field function was used to enable an accurate and detailed observation of the damage. The results of this observation are presented in Figure 20, Figure 21 and Figure 22.

**Figure 23:**
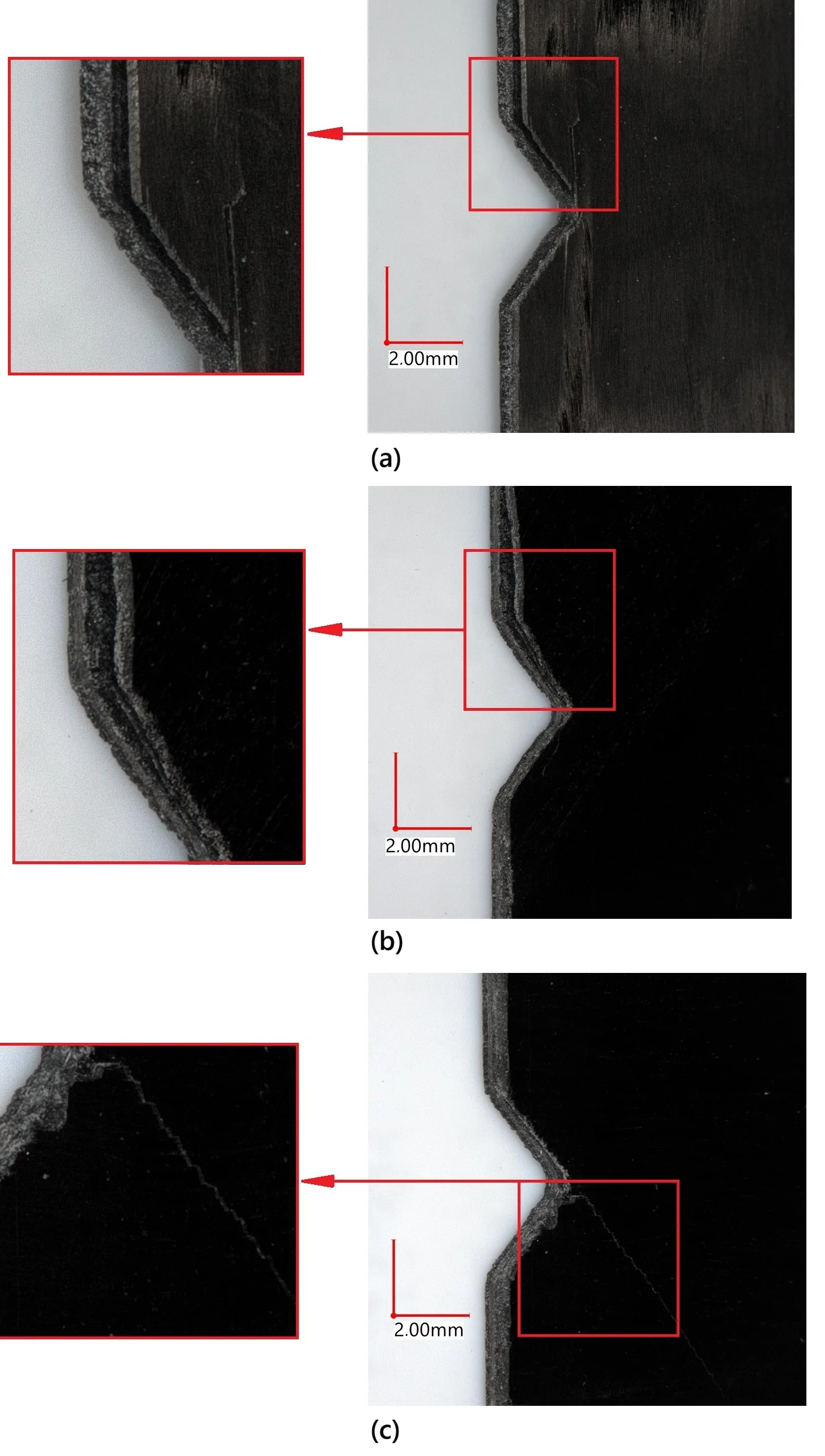
Detailed presentation of types of damage: (**a**) specimen P1-type (P1-1); (**b**) specimen P2-type (P2-1); (**c**) specimen P3-type (P3-1).

The damage phenomenon was assessed based on observations made using digital microscopy. First and most importantly, it was observed that each of the specimens (P1, P2, and P3) showed a different form of damage caused by fatigue testing. Fatigue damage occurred in the notch area of all specimens examined. A characteristic form of damage consisting of fractures in the outer layers forming vertical stripes in the longitudinal direction was observed in all specimens of the first P1-type. The fracture occurred at the tip of the notch. A form of damage consisting of delamination of the outer layers (without layer fractures), which progressed mainly in the longitudinal direction, was observed in all specimens of the second P2-type. The depth of the delamination was similar to the width of the notches. A form of damage consisting of fractures in the outer layers propagating from the centre of the notch at a 45° to the central part of the sample was observed in all specimens of the third P3-type. Furthermore, it was observed that all specimens demonstrated a certain common feature, regardless of the type of damage. All forms of damage occurred in a characteristic manner involving damage within the notches occurring diagonally relative to the vertical axis of symmetry of the test specimens. Consequently, if damage propagated in a certain specimen, for example, upwards from the left notch, then in the case of the right notch, it propagated downwards. Regardless of the type of damage observed, propagation of damage in the notches occurred in the opposite direction. Figure 23 shows the damage forms obtained in detail.

**Figure 24:**
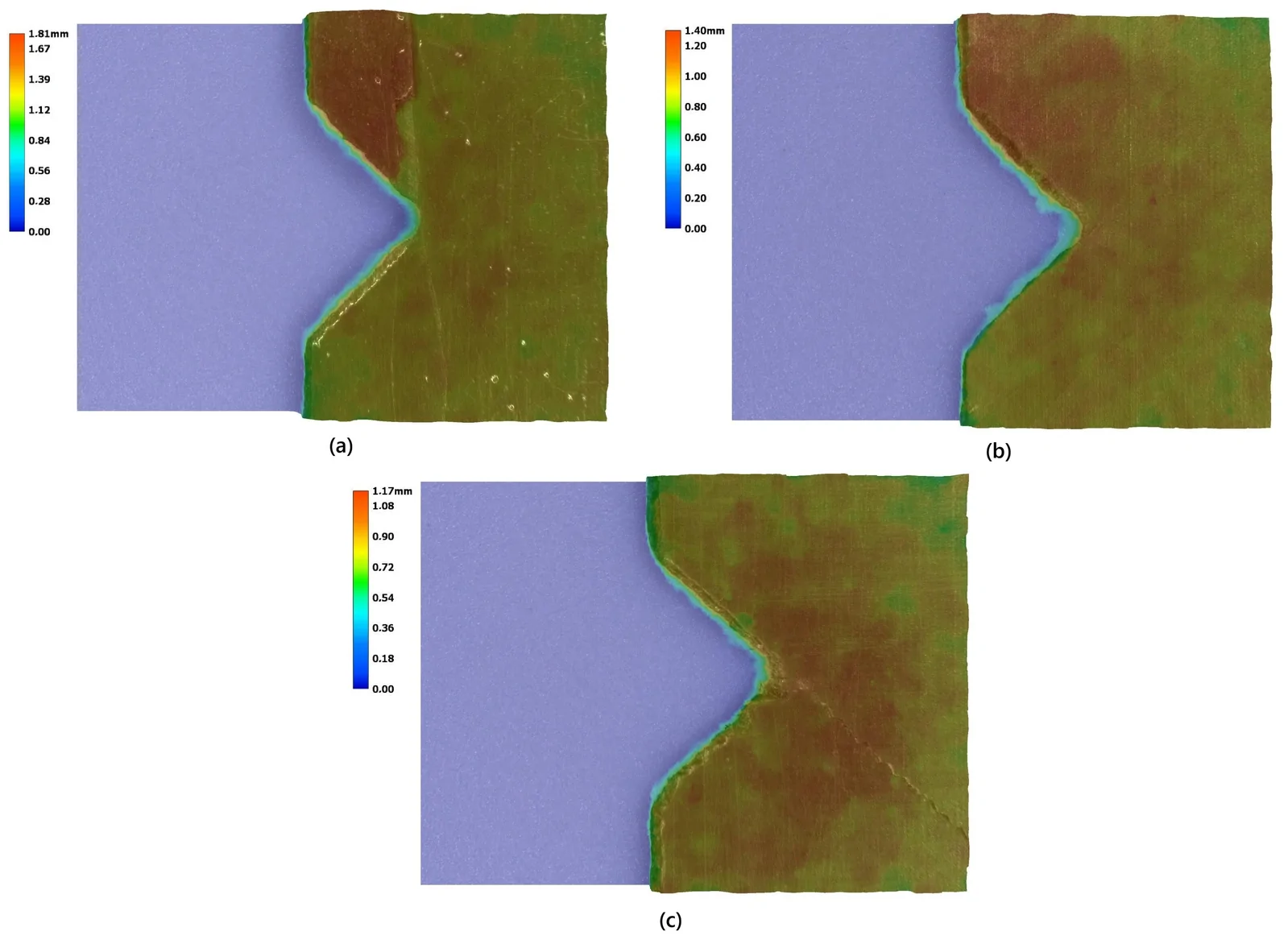
3D visualization of the damage area: (**a**) P1-type; (**b**) P2-type; (**c**) P3-type.

The brightness enhancement and contrast reduction functions (graphics options) were used to highlight the damage during the research. In all tested specimens, the following settings were used to highlight the damage: +20% brightness, −40% contrast. The application of identical corrections within the graphic settings resulted in different shades of colour (black) in the outer layers of all specimens. This was because the angle of the fibres in the outer layers of each type of test specimen was different, which affected the laminate’s colour shade. For P1 specimens, the outer layers had fibres arranged at a 0° angle (light shade of laminate colour), whereas for P2 and P3 specimens, the outer layers had fibres arranged at 45° and 90° angles, respectively (dark shade of laminate colour). However, the use of the above settings was necessary to capture the damage phenomenon. Microscopic analysis demonstrated a significant influence of the composite lay-up on fatigue damage for each test specimen type. Additional microscopic analyses were performed for each type of test specimen (P1, P2, and P3), including 3D visualisations of the damaged areas. Figure 24 shows 3D visualisations of damage forms with depth of field applied.

For P1-type specimens, fractures occurred at the notches, resulting in the formation of vertical (detached) stripes (i.e., fracture of the laminate’s outer layers). P2-type specimens showed delamination, while P3-type specimens showed diagonal fractures propagating from the notch. The legend in Figure 24 illustrates the change in displacement within the analysed parts of the specimens in the normal direction (i.e., perpendicular to the specimen surface). This made it possible to highlight the most damaged areas of the structure (shown in red). The recorded result of the displacement change includes the thickness of the test specimens.

## 4. Discussion and Summary

The differences in damage morphology observed for the investigated stacking sequences can be interpreted in terms of the anisotropic mechanical response of individual plies and the redistribution of stresses within the laminate. The orientation and position of each ply determine its contribution to the overall stiffness and influence the compatibility of deformation between adjacent layers. Consequently, changes in stacking sequence may alter the bending response, extension–bending coupling, and local interlaminar stress distribution, depending on the loading conditions.

Matrix cracking is primarily associated with transverse tensile stresses and local stress concentrations within individual plies. The different directional stiffnesses of adjacent plies can constrain local deformation and influence both crack initiation and subsequent propagation. When a matrix crack reaches a ply interface, its further development depends on the local stress state and the relative fracture resistance of the intralaminar and interlaminar paths. The crack may therefore be arrested, deflected, or act as an initiation site for delamination.

Delamination is governed by the combined effects of interlaminar normal and shear stresses and the fracture resistance of the interface. Interfaces between plies with different fibre orientations may experience localised stress concentrations because the adjacent plies respond differently to the applied loading. In addition, matrix cracks and other intralaminar defects can promote delamination by increasing the local driving force for interfacial crack growth. The observed delamination path should therefore be interpreted in relation to the location of the interfaces, the local stress state, and the resistance to crack propagation under the prevailing loading mode.

Fibre fracture occurs when the local longitudinal stress in the fibres exceeds their strength. The orientation of the fibres relative to the applied load determines their contribution to load carrying, while the surrounding plies influence the redistribution of load following local fibre failure. This interaction may affect the development of additional matrix cracks and delamination, thereby contributing to the differences in damage morphology between stacking sequences.

Three points are worth clarifying: 0° layers typically carry a large portion of the axial load when subjected to tensile stress along the fibres; 90° layers are more susceptible to matrix damage under stresses perpendicular to the fibres; 45° layers play a significant role in transferring shear loads and can influence local stress redistribution.

Overall, the stacking sequence influences the interaction between intralaminar damage and interlaminar crack growth through its effects on laminate stiffness, deformation compatibility, and local stress redistribution. These mechanisms provide a plausible mechanical explanation for the observed differences in crack propagation and delamination behaviour. However, quantitative confirmation of the local stress fields would require additional stress analysis or finite element modelling. Accordingly, the proposed interpretation is based on established laminate mechanics and should be considered in conjunction with the experimental observations.

The results of the present study were similar to previous findings [66]. The previous publication addressed the analysis of how various types of notches affect the structure’s fatigue response and the results of non-destructive testing. The results of this study and the previously published research provide a basis for evaluating relationships related to specimen stiffness degradation, temperature rise characteristics, and forms of damage.

During low-cycle fatigue testing, the initial signs of damage in the composite material were identified through both qualitative and quantitative analyses. The fatigue testing procedure enabled, among other parameters, the progressive reduction in the stiffness of the composite specimens to be evaluated as a function of the applied number of load cycles. The results (Table 2) demonstrated that the composite lay-up had a significant influence on the rate and extent of stiffness degradation during cyclic loading. The decrease in stiffness was as follows: 6.42% for P1 specimens, 6.58% for P2 specimens and 3.48% for P3 specimens. Preliminary signs of damage were assessed in the case of AE testing. It was observed that, for P1 specimens, the recorded energy signal exhibited the highest individual ‘peaks’ at approximately 200–300 pJ. For P2 specimens, the local maximum energy signal peaks were in the range of 100–200 pJ. In the case of P3 specimens, the local energy peaks were characterised by low values at a level of several pJ. Furthermore, the use of wavelet transform made it possible to present certain changes related to the acoustic emission signal in terms of the frequency-time characteristics. The most significant changes related to the damage phenomenon occurred at a frequency of approximately 150 kHz, regardless of the composite materials’ layer arrangement. The intensity of the progressive changes was similar for specimens of different types. In the case of passive thermography, it was observed that the composite layup had a significant impact on both the temperature increase parameter and the form of damage in the most loaded areas (notches). The highest temperature increases were observed in P2 specimens (max. 15.17 °C), slightly lower in P3 specimens (max. 13.84 °C) and lowest in P1 specimens (max. 9.28 °C). A relatively constant increase in temperature was observed in the area between the notches. An irregular increase in temperature was observed in the notches due to damage occurring there. In the context of qualitative assessment, passive thermography and digital microscopy were used to observe similar forms of damage to the specimens (enabling the detailed presentation of these forms of damage). It was observed that the composite lay-up had a very significant influence on the form of damage during fatigue testing. The types of damage observed in the specimens that were tested are as follows: fracturing of the outer layers forming vertical stripes in the area of notches—specimens P1; delamination in the area of notches—specimens P2; fractures of the outer layers at an angle of 45° beginning at the tips of the notches—specimens P3. The independent use of a variety of non-destructive experimental testing techniques made it possible to assess the damage to the composite specimens. The initial indications of damage during low-cycle fatigue testing were analysed, and the effect of the composite lay-up on fatigue strength was assessed. Further research will involve the additional use of FEM simulations.

The results should be interpreted as experimental trends rather than conclusive evidence of statistically significant effects of stacking sequence. Due to the limited sample size, additional statistical analyses and error bars were not introduced, as their inferential value would be limited. Further experiments involving a larger number of specimens are required to assess repeatability and strengthen the statistical robustness of the comparisons.

## 5. Conclusions

The strength of composites was assessed based on experimental research under low-cycle fatigue testing conditions. The test set-up, which consisted of a fatigue machine, passive thermography, acoustic emission, as well as digital microscopy, enabled the damage phenomenon to be thoroughly assessed during the experimental tests. The following conclusions were derived from experimental research conducted using several complementary and independent methods:

- The greatest decrease in structural stiffness was observed in specimens of P1 and P2 types—6.42% and 6.58%, respectively, while the smallest decrease (3.48%) was observed in P3 type specimens;
- The application of AE monitoring during fatigue strength testing enabled the detection of the earliest localised signs of damage within the composite material. The frequency of the measured signal was approximately 150 kHz, regardless of the type of damage identified;
- The values of the highest acoustic emission peaks strongly depend on the configuration of the laminates. The highest peaks were observed for P1-type specimens (200–300 pJ) and slightly smaller for P2-type specimens (100–200pJ). In the case of P3-type specimens, the highest peaks were two times smaller;
- The degradation process was multi-stage and mainly involved matrix cracking, delamination, and interlaminar damage (based on WT);
- The use of passive thermography enabled the assessment of temperature (gradient) increase during fatigue tests—a quantitative aspect, and the estimation of fatigue damage areas—a qualitative aspect;
- The highest temperatures were registered for P2- and P3-type specimens—15.17 °C and 13.84 °C, respectively. In the case of P1-type specimens, the highest temperatures were significantly smaller (9.28 °C);
- A qualitative assessment of various forms of damage depending on the composite lay-up was enabled by the use of digital microscopy;
- During fatigue testing, it was observed that damage initiation began at the tip of the notches;
- The composite lay-up had a significant influence on the form of damage phenomenon that was observed during experimental tests.

## Figures and Tables

**Table 1:** Mechanical properties of tested carbon fibre reinforced polymer composite material.

Mechanical Parameters		Strength Parameters	
Young’s modulus *E*_1_ 0° (MPa)	143,528.5	Tensile strength *F*_TU_ 0° (MPa)	2220.7
Young’s modulus *E*_2_ 90° (MPa)	5826.3	Tensile strength *F*_TU_ 90° (MPa)	49
Poisson’s ratio *ν*_12_	0.36	Compression strength *F*_CU_ 0° (MPa)	641
Kirchhoff’s modulus *G*_12_ (MPa)	3845.5	Compression strength *F*_CU_ 90° (MPa)	114
		Shear strength *F*_SU_ ± 45° (MPa)	83.5

**Table 2:** Summary with critical measured parameters and damage types.

Specimen Group	Decrease in Structural Stiffness *K*	AE Highest Peaks Range	Frequency of Acoustic Emission Signal	Highest Temperature	Damage Type ^1^
P1	6.42%	200–300 pJ	appr. 150 kHz	9.28 °C	FRA
P2	6.58%	100–200 pJ	appr. 150 kHz	15.17 °C	DEL
P3	3.48%	5–7 pJ	appr. 150 kHz	13.84 °C	FRA

^1^ FRA—fracturing of the outer layers in the area of notches; DEL—delamination in the area of notches.

## Data Availability

The original contributions presented in this study are included in the article. Further inquiries can be directed to the corresponding author.
